# Discovery of a High Affinity Adenosine A_1_/A_3_ Receptor Antagonist with a Novel 7-Amino-pyrazolo[3,4-*d*]pyridazine Scaffold

**DOI:** 10.1021/acsmedchemlett.2c00052

**Published:** 2022-05-31

**Authors:** Anna Suchankova, Margarita Stampelou, Klontiana Koutsouki, Athanasios Pousias, Lakshiv Dhingra, Kerry Barkan, Nicole Pouli, Panagiotis Marakos, Roxane Tenta, Antonios Kolocouris, Nikolaos Lougiakis, Graham Ladds

**Affiliations:** § Laboratory of Medicinal Chemistry, Section of Pharmaceutical Chemistry, Department of Pharmacy, School of Health Sciences, National and Kapodistrian University of Athens, Panepistimiopolis-Zografou, 15771 Athens, Greece; ⊥ Department of Pharmacology, University of Cambridge, Tennis Court Road, Cambridge CB2 1PD, U.K.; ∇ Department of Nutrition & Dietetics, School of Health Sciences and Education, Harokopio University, 17671 Athens, Greece

**Keywords:** Adenosine A_1_ receptor, adenosine A_3_ receptor, adenosine A_2B_ receptor, antagonist, binding kinetics, BRET, cAMP, cytotoxicity, molecular dynamics, mutagenesis, residence time

## Abstract

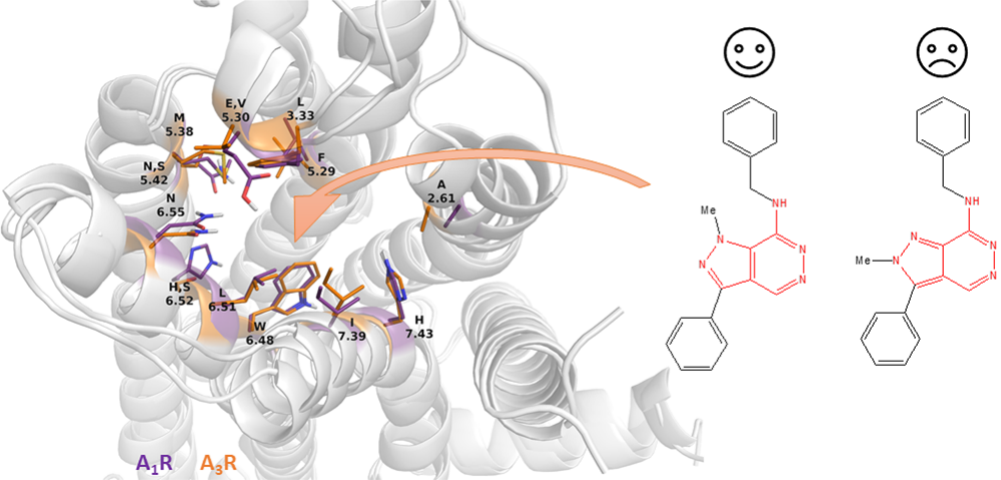

Here we describe the design and synthesis of pyrazolo[3,4-*d*]pyridazines as adenosine receptor (AR) ligands. We demonstrate that the introduction of a 3-phenyl group, together with a 7-benzylamino and 1-methyl group at the pyrazolopyridazine scaffold, generated the antagonist compound **10b**, which displayed 21 nM affinity and a residence time of ∼60 min, for the human A_1_R, 55 nM affinity and a residence time of ∼73 min, for the human A_3_R and 1.7 μΜ affinity for the human A_2B_R while not being toxic. Strikingly, the 2-methyl analog of **10b**, **15b**, had no significant affinity. Docking calculations and molecular dynamics simulations of the ligands inside the orthosteric binding area suggested that the 2-methyl group in **15b** hinders the formation of hydrogen bonding interactions with N^6.55^ which are considered critical for the stabilization inside the orthosteric binding cavity. We, therefore, demonstrate that **10a** is a novel scaffold for the development of high affinity AR ligands. From the mutagenesis experiments the biggest effect was observed for the Y271^7.46^A mutation which caused an ∼10-fold reduction in the binding affinity of **10b**.

Adenosine, a naturally occurring purine nucleoside, is the endogenous agonist of adenosine receptors (ARs).^1^ ARs are G protein-coupled receptors (GPCRs) comprising four subtypes; A_1_, A_2A_, A_2B_, and A_3_. The A_2A_ and A_2B_ subtypes act synergistically with Gα_s_ stimulating adenylyl cyclase and, therefore, increasing 3′,5′-cyclic adenosine monophosphate (cAMP) levels. In contrast, A_1_ and A_3_ receptor subtypes inhibit adenylyl cyclase and decrease cAMP levels by coupling to the G_i/o_ family of G proteins.

In the last two decades numerous heterocyclic compounds have been synthesized as AR ligands including xanthines and bi- or tricyclic fused heterocyclic analogues, e.g., purines, deazapurines, pyrazolopyridines, imidazotriazines, thienopyridazines, naphthyridines, pyridopyrimidines, and pyrazoloquinolines.^2−4^

Different therapeutic applications have been identified in preclinical and clinical studies for A_1_R antagonists as potassium-sparing diuretic agents with kidney-protecting properties,^2^ treatments for chronic lung diseases such as asthma,^5,6^ and possible use in Parkinson’s disease.^7^

A_3_R has been reported to be overexpressed in several types of cancer cells and is, thus, considered as a biological marker for tumors.^8^ In a recent study, the potent and selective A_3_R antagonist LJ-1888 ((2*R*,3*R*,4*S*)-2-[2-chloro-6-(3-iodobenzylamino)-9H-purine-9-yl]tetrahydrothiophene-3,4-diol) blocked the development and attenuated the progression of renal interstitial fibrosis,^9^ while A_3_R antagonists have demonstrated efficacy in eye pathologies by lowering intraocular pressure.^10^

While the binding mode of several agonists and antagonists at A_1_R has been revealed with X-ray crystallography or cryogenic electron microscopy,^11−13^ the experimental structures for A_3_R and A_2B_R have, to date, not been resolved, and only homology models can be used for these AR subtypes.

By the repurposing of antiproliferative aromatic condensed nitrogen heterocycles, we previously identified nanomolar affinity pyrazolo[3,4-*c*]pyridine A_1_R/A_3_R antagonists.^14^ It has been reported that non-xanthine pyrazolo derivatives that potently bind ARs are pyrazolo[4,3-*d*]pyrimidines,^3^ pyrazolo[1,5-*c*]quinazolines,^15^ pyrazolo[3,4-*b*]pyridines,^16,17^ pyrazolo[3,4-*b*]pyridines, pyrazolo[4,3-*e*]-1,2,4-triazolo[1,5-*c*]pyrimidines, pyrazolo[3,4-*c*]- or -[4,3-*c*]quinolines, pyrazolo[4,3-*d*]pyrimidinones, pyrazolo[3,4-*d*]pyrimidines, and pyrazolo[1,5-*a*]pyridines.^18^ After we previously identified the potent pyrazolo[3,4-*c*]pyridine A_1_R/A_3_R antagonists^14^ and observed that certain substituted pyrazolo[3,4-*b*]pyridines had antagonistic potency against A_3_R or A_1_R,^16,17^ we quantified the novel pyrazolo[3,4-*d*]pyridazine scaffold for activity at ARs. Here, we synthesized a series of new 3-alkyl- or 3-aryl-7-amino-pyrazolo-[3,4-d]pyridazine derivatives and determined their affinities against the different ARs using functional cAMP accumulation assays, fluorescent ligand displacement binding studies, and molecular dynamics (MD) simulations.^19,20^ We identified the 21 nM A_1_R/55 nM A_3_R/<2 μΜ A_2B_R antagonist 1-methyl-3-phenyl-7-benzylaminopyrazolo[3,4-*d*]pyridazine (**10b**) as a lead compound. Strikingly, compound **15b**, the 2-methyl congener of **10b**, had lower affinity by >100-fold against 3AR subtypes since, we assumed, it cannot form hydrogen bonding interactions with N^6.55^ which are considered critical for stabilization inside the orthosteric binding cavity. Finally, as these new compounds present structural similarity to antiproliferative purine analogues,^21^ we evaluated their cytotoxic potential against the human fibroblasts cell line (WI-38) and prostatic (PC-3) and colonic (HCT116) cancer cell lines.

## Similarity Calculations

Searching the CHEMBL^22^ database to determine if pyrazolo[3,4-*d*]pyridazine has been used as a scaffold for ligands binding to ARs, using a TanimotoCombo (Tc)^23^ coefficient > 0.85, we did not find any pyrazolo[3,4-*d*]pyridazine derivatives with potency against ARs, suggesting that it is a novel ring system for the development of AR ligands. When we considered the amide 7-benzylamino-3-phenylpyrazolo[3,4-*d*]pyridazine, we found the 4-(2-phenethyl)amino 1-phenylethylpyrazolo[3,4-*b*]pyridine (T_c_ = 0.15) had been reported to bind A_1_R.^16,17^ Thus, we proceeded with a structural activity relationship study around 7-benzylamino-3-phenyl pyrazolo[3,4-*d*]pyridazine and synthesized a series of 7-amino-pyrazolo[3,4-*d*]pyridazines for biological evaluation against ARs.

## Chemistry

**Scheme 1:**
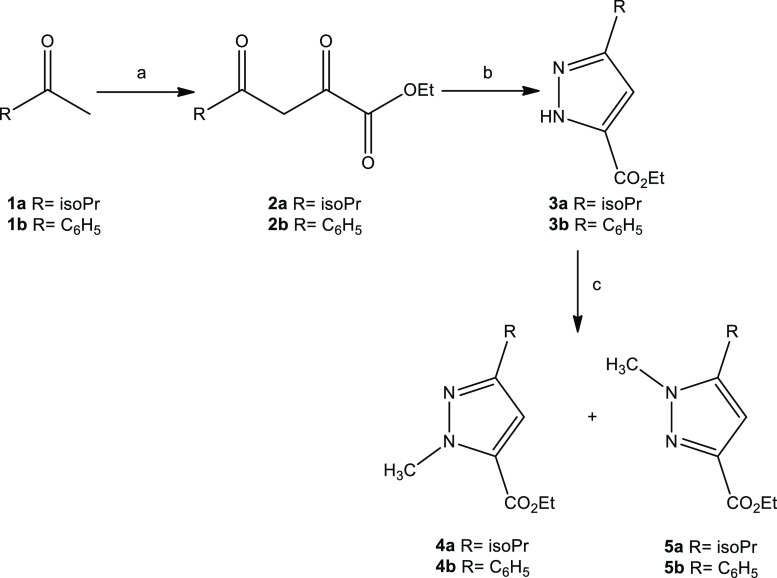
Synthesis of **4a,b** and **5a,b** Reagents and conditions: (a) diethyl oxalate, NaH 60%, toluene dry, 50°C, 2 h; (b) NH_2_NH_2_ 80%, EtOH, reflux, 90 min; (c) (i) NaH 60%, DMF dry, 0 °C, 15 min, (ii) CH_3_I, rt, 1 h.

The synthesis of the target compounds was accomplished through the previously reported pyrazolecarboxylates **4a,b** and **5a,b** (Scheme 1). Briefly, commercial isopropylmethylketone (**1a**) or acetophenone (**1b**), was first converted to the ethyl 2,4-diketocarboxylates **2a** and **2b**, respectively,^24,25^ which upon reaction with hydrazine monohydrate gave the pyrazolecarboxylates **3a,b**.^26^ These were methylated using methyl iodide in the presence of sodium hydride and provided the regioisomers **4a,b**(27,28) and **5a,b**,^28^ respectively. Interestingly, when we used tetrahydrofuran as solvent in the place of dimethylformamide (DMF), we exclusively obtained the *N*^*1*^-methyl-5-carboxylate **4a** isomer.

**Scheme 2:**
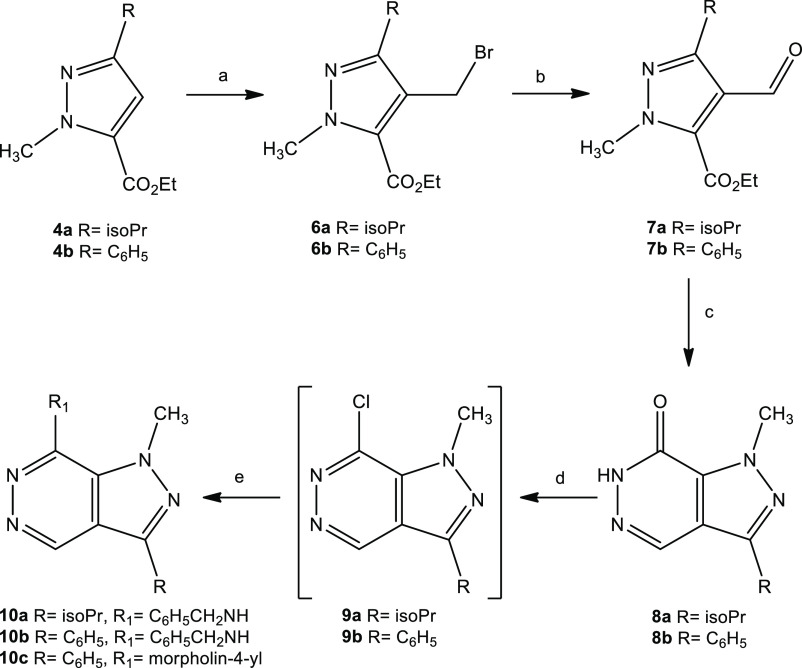
Synthesis of **10a–c** Reagents and conditions: (a) paraformaldehyde, 33% HBr in AcOH, 90 °C, 3.5 h; (b) *Ν*-methylmorpholine-*Ν*-oxide, MeCN dry, rt, 24 h; (c) NH_2_NH_2_ (80%), HCl 36%, EtOH, 90°C, 1 h; (d) POCl_3_, 110 °C, 2.5–8 h; (e) HNR_1_R_2_, EtOH, reflux, 2 h.

**Scheme 3:**
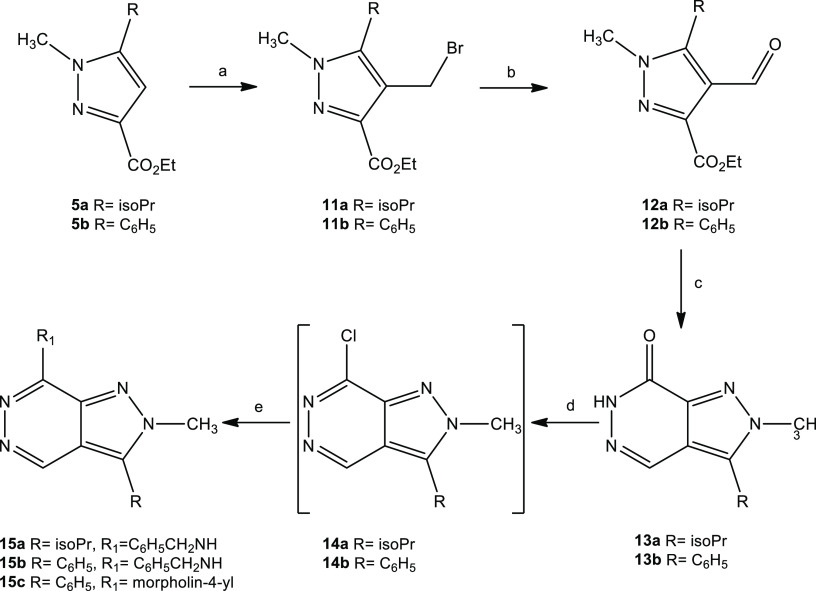
Synthesis of **15a–c** Reagents and conditions: (a) paraformaldehyde, 33% HBr in AcOH, 90 °C, 3.5 h; (b) *Ν*-methylmorpholine-*Ν*-oxide, MeCN dry, rt, 24 h; (c) NH_2_NH_2_ (80%), HCl 36%, EtOH, 90 °C, 1 h; (d) POCl_3_, 110 °C, 2.5–8 h; (e) HNR_1_R_2_, EtOH, reflux, 2 h.

Each of the isomeric pyrazoles **4a,b** or **5a,b** was subsequently treated with paraformaldehyde in the presence of a 33% HBr solution in acetic acid and was converted to the bromides **6a,b** (Scheme 2) or **11a,b** (Scheme 3), respectively. The bromomethyl group was then oxidized using *N*-methylmorpholine *N*-oxide to generate the carbaldehydes **7a,b** (Scheme 2) and **12a,b** (Scheme 3).

The aldehydes **7a,b** and **12a,b** were then treated with hydrazine, and upon ring closure the pyrazolopyridazinones **8a,b** and **13a,b** were obtained. The pyridazinones reacted with phosphorus oxychloride to give the corresponding chloro derivatives **9a,b** and **14a,b** with suitable purity that they could be introduced to the next reaction. These crude products were then treated with benzylamine or morpholine to result in the target compounds **10a**–**c** and **15a**–**c** (Figures S1–S3).

## Assessing Biological Activity of Pyrazolo[3,4-*d*]pyridazine Derivatives

### cAMP Assays Assessing Activity at Adenosine Receptors

**Figure 1:**
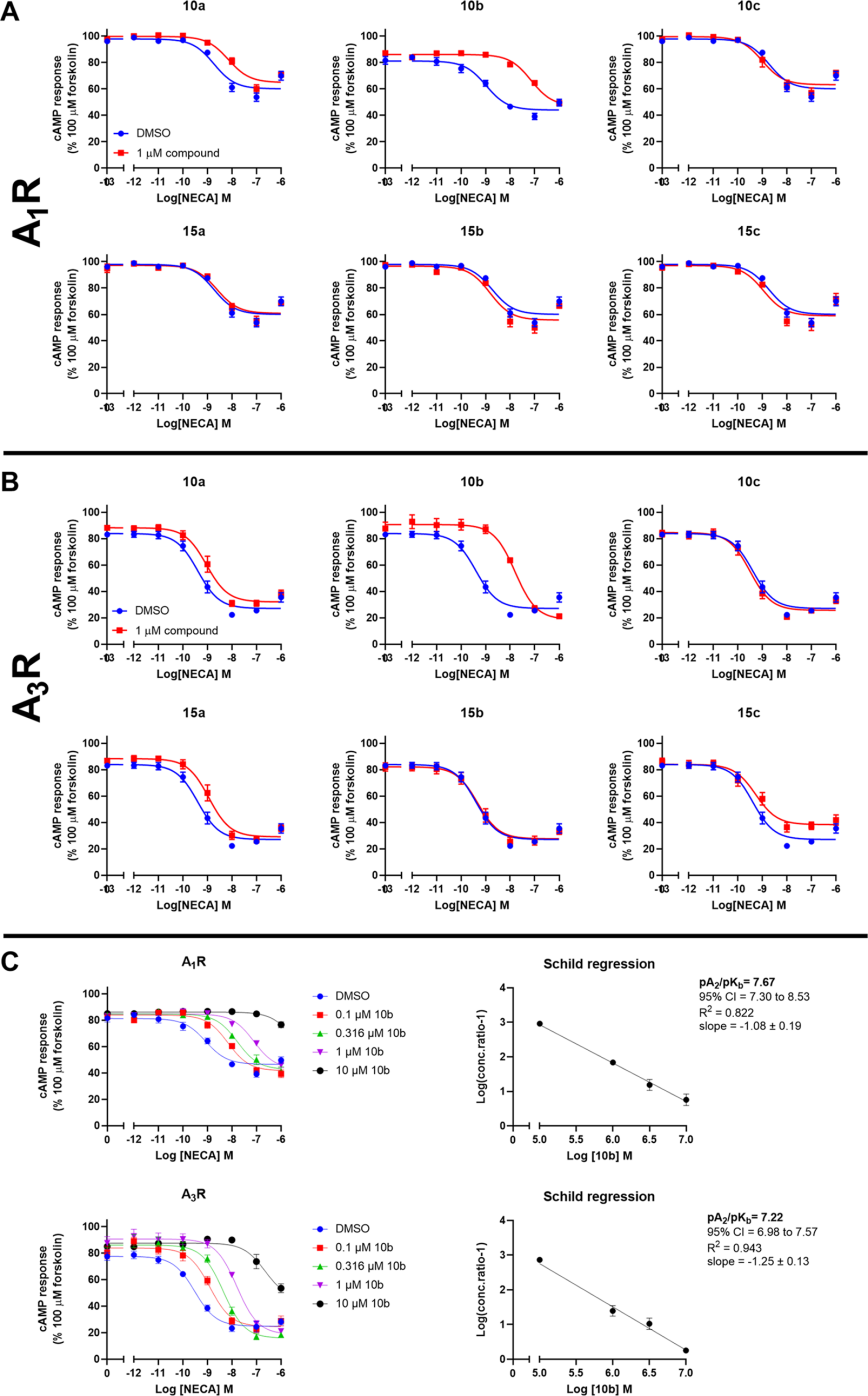
Characterization of 7-amino-pyrazolo[3,4-*d*]pyridazines at human A_1_R and A_3_R. (A and B) Cells expressing either human A_1_R (A) or A_3_R (B) were exposed to 10 μM forskolin and stimulated with increasing concentrations of NECA for 30 min in the presence of a 1 μM concentration of the test compound, and the cAMP accumulation was quantified. (C) cAMP accumulation was measured as detailed in part A using multiple concentrations of **10b**. Using pEC_50_ values, Schild regression analysis was conducted to calculate pA_2_/p*K*_b_ values. All values are mean ± SEM expressed as percentage forskolin inhibition, relative to NECA. *n* ≥ 3 independent experimental repeats were performed in duplicate.

Having synthesized compounds **10a**–**c** and **15a**–**c**, we next tested their activity, as antagonists, against the different human AR subtypes using a single high concentration of the compound (1 μM) coadministered with NECA (5′-*N*-ethylcarboxamidoadenosine) in a cAMP accumulation assay (Figure 1A and B). Note that for A_1_R and A_3_R 10 μM forskolin was added since these are G_i/o_-coupled receptors and reduce cAMP accumulation.^19,29^ All compounds lacked efficacy at NECA-stimulated A_2A_R (even when tested at 10 μM) (Table S1). Compounds **10c**, **15b**, and **15c** also lacked efficacy at the other 3AR subtypes, with **15a** displaying weak efficacy only at A_3_R, while compounds **10a** and **10b** displayed activity at all 3ARs although this was only detectable for A_2B_R when a 10 μM concentration of the compound was used (Table S1). Based upon a single concentration of antagonist, we calculated the equilibrium dissociation constant (p*K*_d_) of each compound (Table 1). Of the compounds tested, **10b** displayed the highest affinity at the different AR subtypes with greater selectivity toward A_1_R and A_3_R than A_2B_R. We next performed a more extensive Schild analysis using multiple doses of the most potent antagonist, **10b**, only at A_1_R and A_3_R (Figure 1C). In both cases **10b** acted as a competitive antagonist, generating a Schild slope that did not significantly differ from unity. Using the Schild plot, we calculated **10b**’s affinity (pA_2_/p*K*_b_) to be 21 nM at A_1_R and 55 nM at A_3_R while only 1.7 μΜ at A_2B_R (Table S1).

**Table 1:**
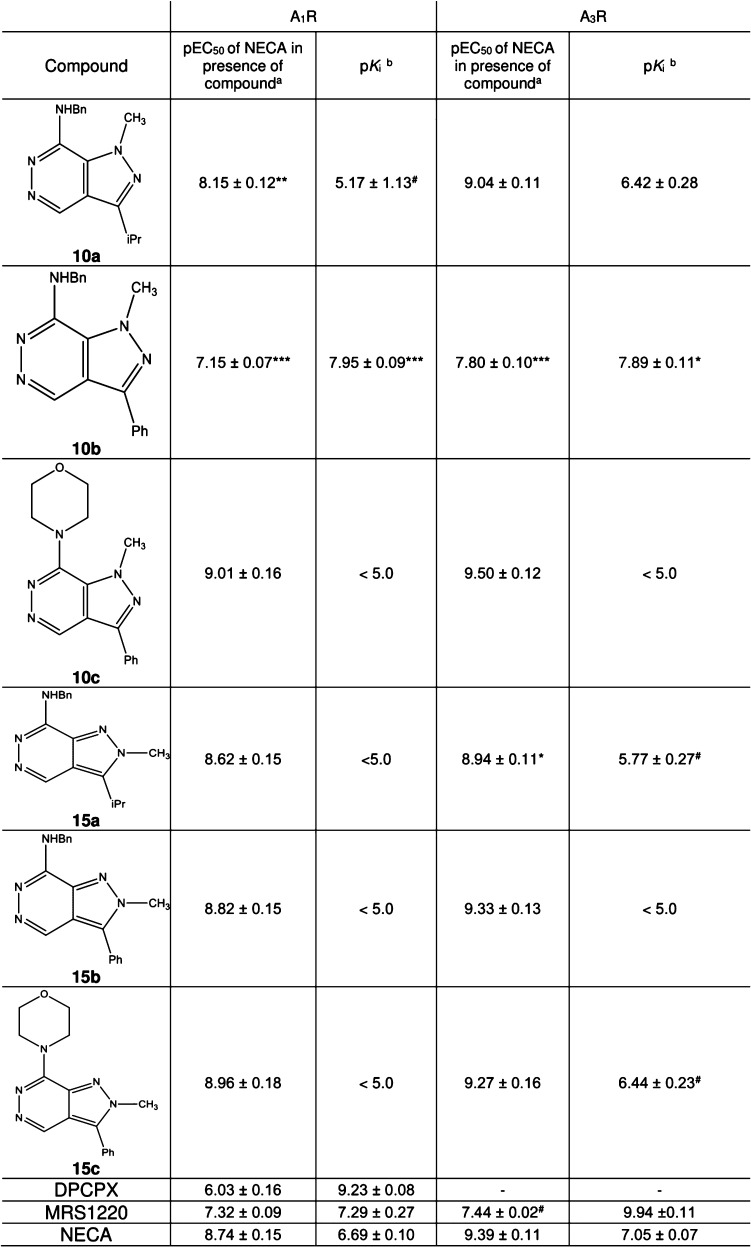
Chemical Structures, Antagonistic Potencies (pEC_50_ in the Presence of NECA^a^), and Affinities (p*K*_i_^b^) of 7-Amino-pyrazolo[3,4-*d*]pyridazines **10a–c** and **15a–c** against A_1_R and A_3_R

a Mean ± SEM; functional activities (pEC_50_ values of NECA in the presence of either 1 μM ligands or vehicle) as mean ± standard error of the mean (SEM) of at least three independent repeats, conducted in duplicate—values obtained from Figure 1.

b Mean ± SEM; equilibrium binding affinities of the ligands measured with NanoBRET against Nluc-A_3_R or Nluc-A_1_R; NECA was used as positive control.^3^

# Due to the high affinity of MRS1220, 10 nM was used to enable measurement of the full dose–response curve of NECA to determine pEC_50_.

Statistical significance compared to NECA was determined, at *p* < 0.05, through one-way ANOVA with Dunnett’s post-test (*, *p* < 0.05; **, *p* < 0.01; ***, *p* < 0.001; ****, *p* < 0.0001).

### Quantifying Binding Parameters Using a NanoBRET-Based Saturation Binding Assay

**Figure 2:**
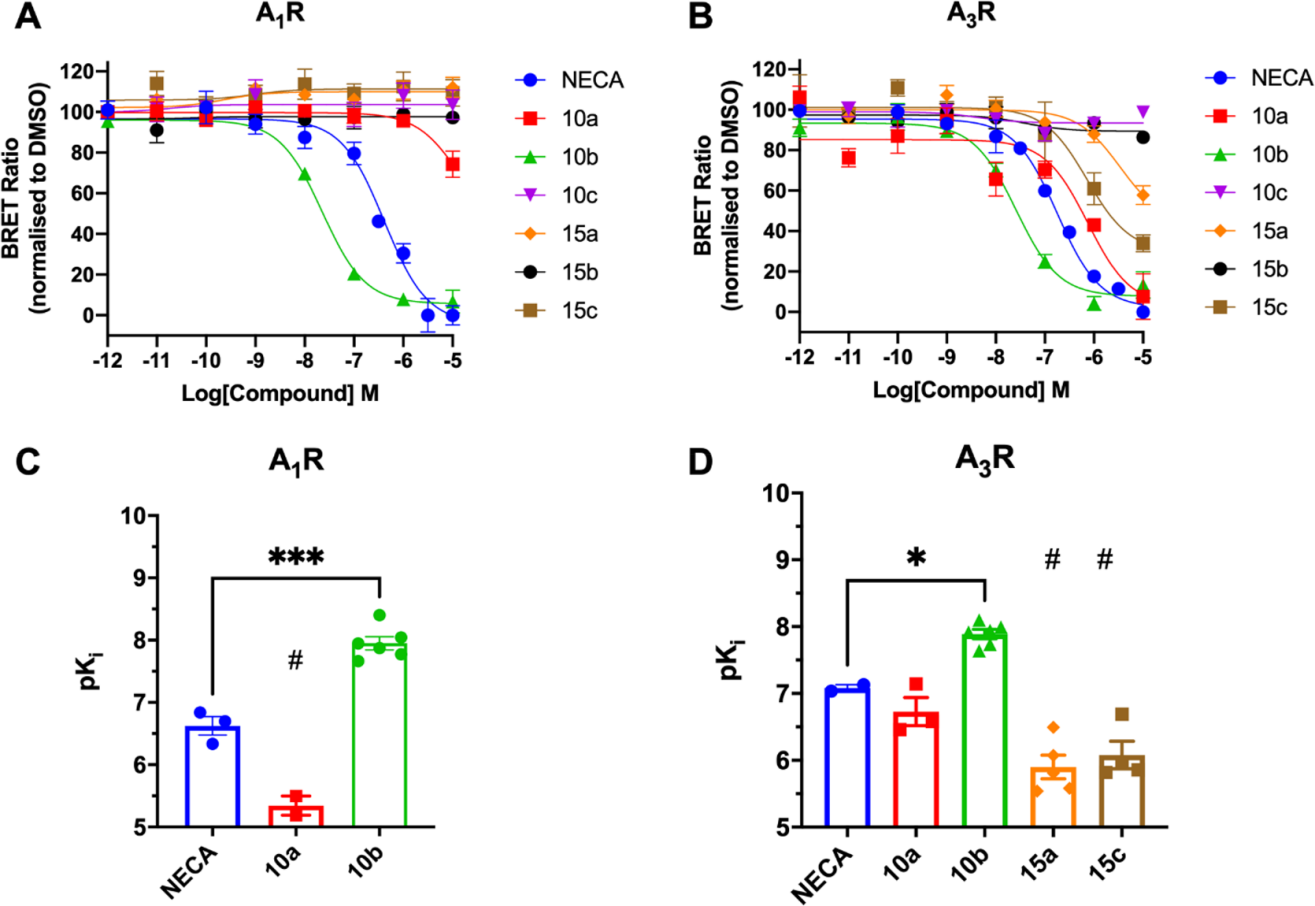
Inhibition of BRET between CA200645 at NLuc-A_1_R and Nluc-A_3_R by **10b** and **10a**. HEK293 cells expressing Nluc-A_1_R (A) or Nluc-A_3_R (B) were treated with 5 nM or 20 nM CA200645, respectively, enabling concentration-dependent decreases in the BRET ratio at 10 min to be determined with the response normalized to DMSO. Binding curves were fitted with the Cheng Prusoff equation built into GraphPad Prism 9.3 to enable estimates of the p*K*_i_.^19^ Comparison of p*K*_i_ values for A_1_R (C) and A_3_R (D) as determined via BRET binding. Each data point represents the mean ± SEM of at least three experiments performed in duplicate. The statistical significance compared to NECA was determined, at *p* < 0.05, through one-way ANOVA with Dunnett’s post-test (*, *p* < 0.05; ***, *p* < 0.001). ^#^Compounds did not fully displace CA200645, so p*K*_i_ values are estimates preventing statistical analysis.

We next sought to independently verify the affinities determined using the Shield analysis by directly quantifying the potential antagonists’ binding to A_1_R and A_3_R using a previously described saturation nano-bioluminescence resonance energy transfer (NanoBRET) binding assay.^19^ We determined the ability of all the compounds to displace the specific binding of CA200645,^30^ a fluorescent antagonist of A_3_R and A_1_R, using Nluc-A_3_R expressing human embryonic kidney 293 (HEK293) and Nluc-A_1_R HEK293 cells (Figure 2 and Table 1). A_2B_R was not included in this analysis since the p*K*_d_ values of **10a** and **10b** at A_2B_R were estimated to be below 1 μM (Figure 1 and Table 1). Consistent with the Schild analysis, compound **10b** displayed the highest affinity at A_1_R and A_3_R (A_1_R, p*K*_i_ = 7.95 ± 0.09; A_3_R, p*K*_i_ = 7.89 ± 0.11). Of the remaining compounds, **10a** displayed weak affinity at A_3_R (p*K*_i_, 6.42 ± 0.28), which agreed with the Schild regression estimate, but failed to fully displace CA200645 at A_1_R, making an estimate for its affinity unreliable. All the other compounds failed to displace CA200645 at A_1_R or A_3_R except for **15a** and **15c**, which did display some binding at A_3_R but, like **10a**, also failed to fully displace CA200645 at the concentrations tested. Significantly, **15b**, which contains an *N*-methyl substitution to 1-NH and 2-NMe compared to 1-NMe and 2-NH in **10b**, failed to bind either AR subtype.

### Determining Kinetic Parameters of **10b** Binding at A_3_R and A_1_R Using NanoBRET

We next investigated the real-time binding kinetics^19,30^ of **10b** at A_3_R and A_1_R using the NanoBRET binding method. Specifically, we quantified **10b**’s ability to inhibit specific binding of CA200645 to Nluc-A_3_R and Nluc-A_1_R expressed in HEK293 cells. The kinetic parameters for CA200645 binding at Nluc-A_3_R were previously determined as *K*_on_ = 32.5 ± 0.28 × 10^5^ M^–1^ min^–1^ and *K*_off_ = 0.025 ± 0.005 min^–1^ with a p*K*_D_ of 10.11. Conversely the kinetics of CA200645 binding at Nluc-A_1_R were determined as *K*_on_ = 14.5 ± 0.4 × 10^5^ M^–1^ min^–1^, *K*_off_ = 0.023 ± 0.001 min^–1^, and p*K*_D_ = 7.80 ± 0.2 nM.^14^ Applying these parameters into the “kinetics of competitive binding” model built into GraphPad Prism9.0, we were able to provide estimates of the kinetics of binding for **10b** against A_1_R (*K*_on_ = 51.4 ± 0.26 × 10^5^ M^–1^ min^–1^, *K*_off_ = 0.019 ± 0.003 min^–1^ with a p*K*_D_ = 7.46 ± 0.1 and RT = 59.8 ± 12.7 min) and against the A_3_R, (K_on_ = 25.6 ± 0.1 × 10^5^ M^–1^min^–1^, K_off_ = 0.0014 ± 0.002 min^–1^ with a p*K*_D_ = 7.26 ± 0.05 and RT = 72.58 ± 8.8 min). None of the other compounds were analyzed using this method due to their extremely fast K_off_ rates (>min^–1^). For compound **10b** there was an excellent agreement between p*K*_D_ (K_on_*/*K_off_) of the compounds from the kinetics assays and the Schild analysis (pA_2_/p*K*_b_) and fair agreement (∼3.16-fold) with the saturation binding assays (p*K*_i_).

## Simulations

### Investigation of the Binding of the 7-Amino-pyrazolo[3,4-*d*]pyridazines to A_1_R and A_3_R

**Figure 3:**
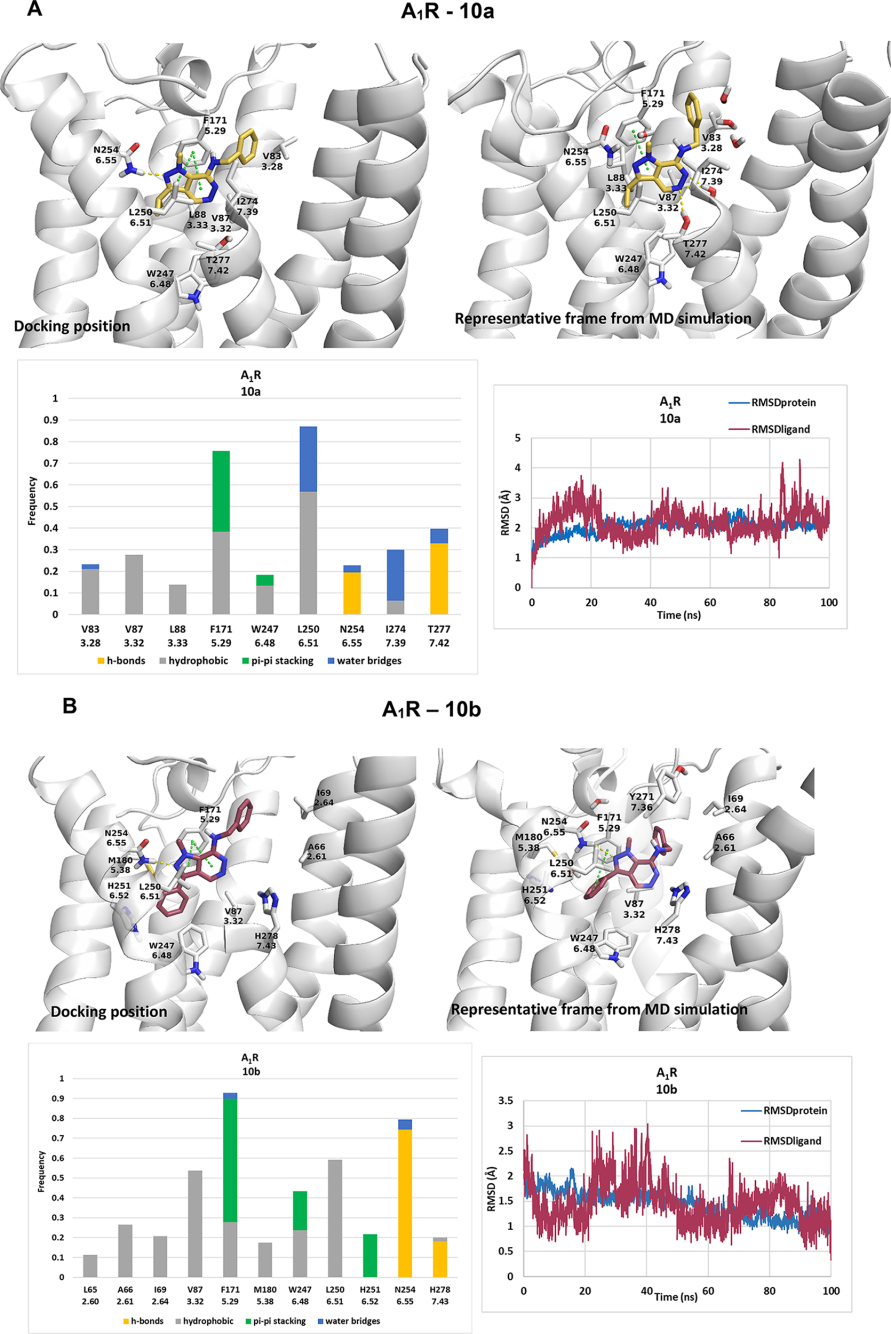

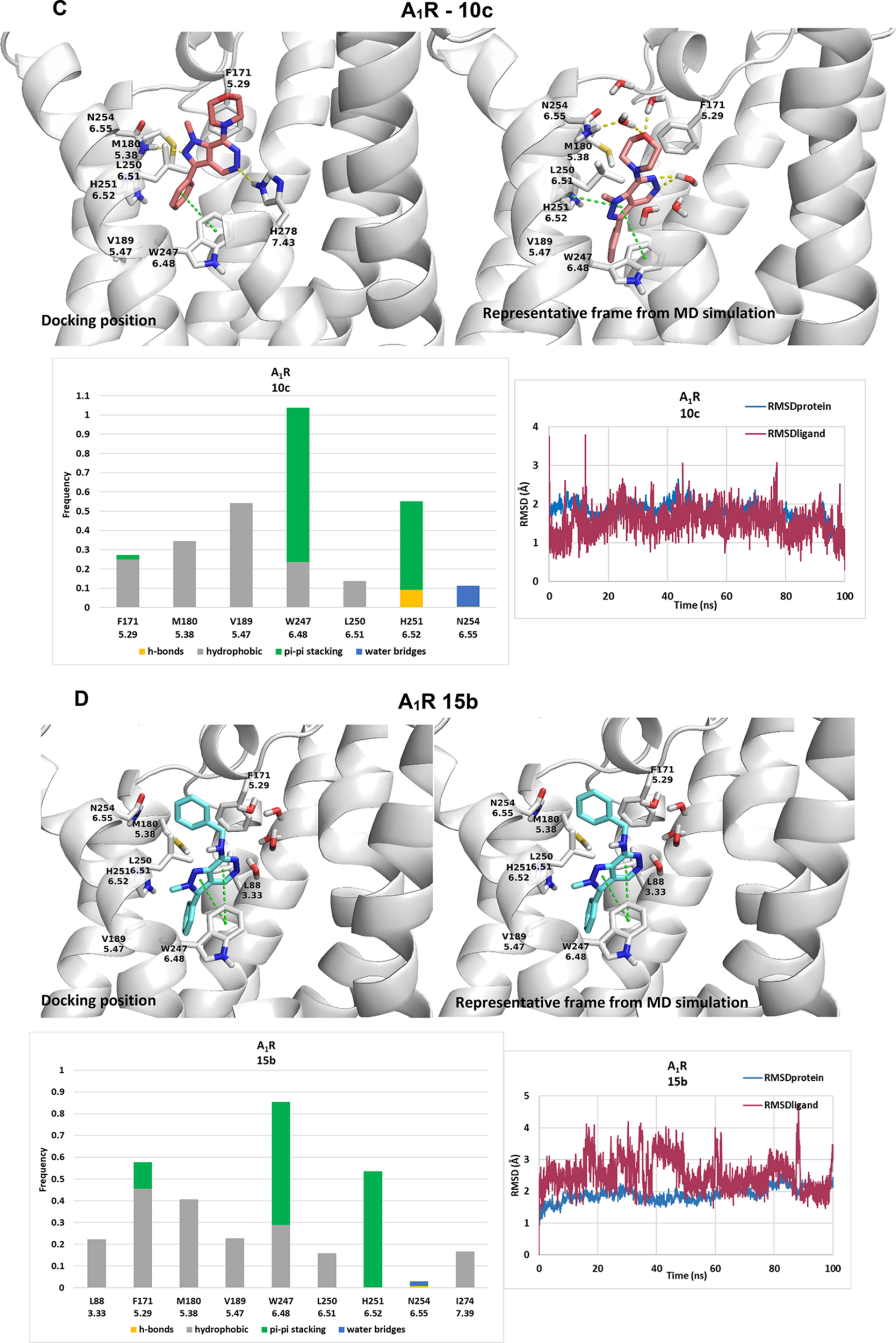
(A–C) 100 ns MD simulations of **10a**–**c** inside the orthosteric binding area of A_1_R. (D) 100 ns MD simulations of **15b** inside the orthosteric binding area of A_1_R. Starting structures are shown (docking pose), and representative frames from MD simulations, receptor–ligand interaction frequency histograms, and RMSD plots of proteins (RMSD_protein_; blue plots) and ligand heavy atoms (RMSD_ligand_; red plots) inside the orthosteric binding area of WT A_1_R or A_3_R. Bars are plotted only for residues with interaction frequencies ≥0.2. Color scheme: ligand = brown sticks, receptor = white cartoon and sticks, hydrogen bonding interactions = yellow (dashes or bars), π–π interactions = green (dashes or bars), hydrophobic interactions = gray, water bridges = blue. For the protein models of A_1_R in complex with **10a**–**c** or **15b**, the experimental structure of the inactive form of A_1_R in complex with an antagonist (PDB ID 5UEN(4)) was used.

Having pharmacologically evaluated the different compounds, we then used molecular docking to provide insights into how they bind to the ARs. We docked **10a**–**10c** into the orthosteric binding site of A_1_R and **10b** and **15b** into A_2B_R and A_3_R (the amino acid sequences of A_1_R, A_3_R, and A_2B_R in the orthosteric binding area are shown in Scheme S1) using ChemScore as the scoring function^31^ with the highest score docking pose being inserted into a hydrated phosphatidylethanolamine bilayer. The complexes were subjected to 100 ns MD simulations with amber99sb,^32^ and then, the MD simulations’ trajectory was analyzed (Table S2). The MD simulations showed that the 7-benzylamino-pyrazolo[3,4-*d*]pyridazine **10b** substituted with *N*^*1*^Me and a 3-phenyl group formed a stable complex with all 3ARs with RMSD_protein_ values <2.1 Å. Starting from the same docking pose of **10b** in A_1_R or A_3_R (Figure 3), the mean frame from MD simulations was close to the starting docking pose in A_1_R (RMSD_lig_ = 1.21 Å) while in A_3_R (Figure S2) the ligand moved considerably into the cleft between the transmembrane (TM)3, TM5, and TM6 helices (RMSD_lig_ = 4.88 Å). Thus, starting from the same binding pose for **10b**, the MD simulations produced two different binding orientations at A_1_R and A_3_R. This is due to the fact that A_1_R has a broader binding area, expanded toward TM1 and TM2, compared to the other ARs, according to the X-ray structures of A_1_R in complex with antagonists.^11,12^ A similar AR ligand reported in the literature is 4-(2-phenethyl)amino 1-phenylethyl pyrazolo[3,4-*b*]pyridine (Tc = 0.15), which binds with a similar docking pose to **10b** to A_1_R.^16^ We also docked a representative adenine derivative (*N*9-methyl,*N*6-benzyl adenine) to A_1_R and found a similar docking pose (Figure S3).

Inside the A_1_R orthosteric site, compound **10b** formed hydrogen bonds through its pyrazole or pyridazine nitrogen donor groups to the amide side chain of N254^6.5^ or the imidazole side chain of H278^7.43^. Furthermore, **10b** was stabilized in the orthosteric binding site through π–π interactions between its pyrazolo[3,4-*d*]pyridazine or phenyl rings with F171^5.29^, H251^6.2^, and W247^6.48^, respectively. The benzylamino group of **10b** oriented toward the widened TM2 area in A_1_R, forming hydrophobic interactions with A66^2.61^ and I69^2.64^. Furthermore, **10b** was found to bind deep in the pocket interacting with V87^3.32^ and W247^6.48^ while 3-phenyl-pyrazole aligned close to the side chains of M180^5.38^ and L250^6.1^ (Figure 3A). In A_3_R, compound **10b** was stabilized through formation of hydrogen bonding interactions with N254^6.5^ and H278^7.43^ and hydrophobic interactions with L90^3.32^, L91^3.33^, F168^5.29^, M177^5.38^, L246^6.1^, and I268^7.39^ (Figure S2B). Finally, the MD simulations for **10b** (Figure S2A) in complex with A_2B_R (Figure S2) show weak hydrogen bond interactions with N254^6.5^.

Pharmacologically, compounds **10b** and **15b** differed considerably in their affinity to the ARs (Figures 1 and 2 and Table 1). Comparing MD simulations for **15b** with **10b** in the orthosteric binding area of A_1_R, A_3_R (and A_2B_R) shows that starting from a similar docking pose, the substitution from *N*^1^ Me and 2-*N*H (found in **10b**) to *N*^1^H and *N*^2^ Me (in **15b**) results in **15b** failing to generate hydrogen bonds with N^6.55^ because of the steric repulsion between 2-methyl and the amide side chain of N^6.55^; for this reason also **15a** and **15c** were inactive (Figure S2). Although many ligands can have similar docking poses, subtle changes in the ligand substitution pattern can result in significant changes in binding, and this can be followed only with MD simulations. Considering the two active compounds, **10b** and **10a**, replacement of the 3-phenyl group (found in **10b**) with a 3-isopropyl group (generating **10a**) results in a remarkable reduction of affinity. This is due to **10a** losing significant π–π interactions with H251^6.2^ and hydrophobic interactions with residues deeper in the binding site, e.g., W247^6.48^, L250^6.1^, and V87^3.32^ (Figure 3). Finally, substitution of **10b**’s 7-benzylamino by the more rigid morpholinyl group (found in **10c**) resulted in reduced affinity to the ARs. The more rigid morpholino group in **10c** repels F171^5.29^, so the ligand rotates and moves to the bottom of the binding area, losing hydrogen bonding interactions with N254^6.5^ and weakening its hydrophobic interaction with critical residues, e.g., F171^5.29^ and L250^6.1^ (Figure 3). With an accuracy of ∼±4 kcal mol^–1^, the MM-GBSA method^33,34^ (Supporting Information) only provides an approximation when applied to structure–activity relationships for analogs in the same series. Nevertheless, the MM-GBSA binding free energy calculations for ligands **10a**–**c** against A_1_R (Table S2), using the OPLS2005 force field^35,36^ with a hydrophobic slab as an implicit membrane model and including the waters in the orthosteric binding area, predicted fairly well the stability of **10a**–**c** in complex with A_1_R with binding free energy values (after neglecting entropy) Δ*G*_eff_ = −94.50, −96.42, and −85.35 kcal mol^–1^.

### Mutagenesis Experiments to Study **10b** Binding to A_1_R

We have previously observed that mutation of residues that do not directly interact with the ligands (e.g., V^5.30^ for A_3_R, which is more than 4 Å apart from the ligand inside the orthosteric binding area) can, through allosteric interactions due to the plasticity of the binding area, significantly affect ligand affinity.^20,21,37^ As such it is not always straightforward to determine the effects of a mutation on affinity properties. Despite this caveat, we next used mutational analysis combined with NanoBRET to determine the important residues required for **10b** binding to A_1_R. The mutation of L250^6.1^A resulted in only a slight reduction of binding affinity for **10b** (Table 2) despite the MD simulations suggesting that the ligand should be close enough to L250^6.1^ to enable hydrophobic interactions. It is possible that residues H251^6.52^ and W247^6.48^ could contribute to the stabilization of **10b** with hydrophobic interactions even if L250^6.1^ is mutated to alanine. It is noteworthy that mutation of E172^5.30^ (which is also more than 4 Å apart from the ligand inside the orthosteric binding area) to alanine also did not significantly change the binding affinity (Table 2). This contrasts with our studies using 3-phenyl-7-anilinopyrazolo[3,4-*c*]pyridines which showed a 1.5-fold decrease in affinity due to the E172^5.30^A mutation.^14^

**Table 2:** Binding Affinities (p*K*_i_) for **10b** Measured Using Saturation NanoBRET Binding with CA200645 as the Fluorescent Tracer against WT A_1_R and Mutant A_1_Rs

A_1_R	p*K*_i_	Effect on affinity
WT	7.68 ± 0.11	baseline
T91^3.36^A	7.68 ± 0.07	no change
E172^5.30^ A	7.34 ± 0.06	no significant change
L250^6.51^ A	7.57 ± 0.04	no significant change
H251^6.52^A	7.62 ± 0.06	no significant change
S267^7.42^A	7.86 ± 0.03	no significant change
Y271^7.46^A	6.99 ± 0.05	∼10-fold reduction

In addition, mutation of H251^6.2^A has been reported to reduce antagonist affinity against A_3_R^20,21^ although here it did not have any effect on **10b** affinity at A_1_R. Other residues of interest to mutate were T91^3.36^A and S267^7.42^A, which are deep in the orthosteric pocket. Interestingly, we found that mutation to alanine of these residues also did not have a significant effect on the binding affinity of **10b** (Table 2). This is in contrast to our results for pyrazolo[3,4-*c*]pyridines which can interact directly with these residues.^9^ The results for **10b** suggested that it is positioned above pyrazolo[3,4-*c*]pyridines,^9^ in the A_1_R pocket, and so unaffected by these mutations.

The biggest effect in this study was observed for the Y271^7.46^A A_1_R mutation, which caused a ∼10-fold reduction in the binding affinity of **10b** (Table 2). This effect is in contrast to that observed previously for pyrazolo[3,4-*c*]pyridines^14^ for which we showed that the Y271^7.46^A mutation caused a slight increase in binding affinity. Since the MD simulations showed contacts with H278^7.43^ and not Y271^7.46^, the Y271^7.46^A mutation in A_1_R might affect the binding of **10b** through contact with H278^7.43^. We performed the MD simulations of **10b** in complex with A_1_R-Y271^7.46^A and observed that the ligand loses its hydrogen bonding interactions with N254^6.5^, which might weaken its binding interactions with the orthosteric binding area (Figure S4).

### Preliminary Toxicological Analysis of Pyrazolo[3,4-*d*]pyridazine Derivatives

Given the high affinity **10b** displays for A_1_R and A_3_R, and thus the potential for it to be a scaffold for future compound development, we wanted to evaluate its antiproliferative nature as an early indicator of its toxicological profile. We therefore evaluated **10b**, alongside the other compounds in this study, for cytotoxic activity against human fibroblasts (WI-38) and two cancer cell lines, namely the prostate cancer (PC-3) and colon cancer (HCT116) cell lines. Importantly, **10b** alongside all the compounds proved to be not cytotoxic against the cell lines, with IC_50_ values >10 μM. The only compound that did display any cytotoxicity was **15b**, which displayed moderate cytotoxicity against the PC-3 and HCT116 cell lines, showing IC_50_ values of 5.3 ± 0.1 μM against PC-3 cells and 4.15 ± 0.05 μM against HCT116 cells. As a result of these data, we are confident that **10b** is noncytotoxic and can be progressed for further development as a dual A_1_R/A_3_R antagonist.

## Supporting Information Available

Scheme S1. Comparison of amino acid residue sequences of the binding area. Table S1. Chemical structures and antagonistic potencies of 7-amino-pyrazolo[3,4-*d*]pyridazines **10a–c** and **15a–c** against A2AR and A2BR. Table S2. Mean RMSD values for all compounds against A_1_R, A_2A_R, and A_2B_R and Δ*G*_eff_ only for **10–10c** against A_1_R. Figure S1. ^1^H and ^13^C NMR spectra of the target compounds. Figure S2. Results from the MD simulations of **10b** and **15b** against A_3_R and A_2B_R. Figure S3. Docking poses of 4-(2-phenethyl)amino-1-phenylethyl pyrazolo[3,4-*b*]pyridine and N9-methyl,N6-benzyl adenine to A_1_R. Figure S4. Representative frames from 100 ns MD simulations of **10b** inside the orthosteric binding area of WT A1R and **10b** inside mutant Y271A A_1_R and the receptor–ligand interaction frequency histogram and RMSD graphs of protein Ca and ligand heavy atoms. Information for the methods and synthetic protocols. (PDF)

## Abbreviations

ARs: adenosine receptors
BRET: bioluminescence resonance energy transfer
GPCRs: G protein-coupled receptors
HEK: human embryonic kidney
MD: molecular dynamics
NECA: 5′-*N*-ethylcarboxamidoadenosine
PDB: Protein Data Bank
RMSD: root-mean-square deviation
T_c_: TanimotoCombo
*t*_m_: mixing time
TM: transmembrane
