# Supplementary material for: Discovery of a High Affinity Adenosine A1/A3 Receptor Antagonist with a Novel 7-Amino-pyrazolo[3,4-d]pyridazine Scaffold
Source: ACS Med Chem Lett. 2022 May 31;13(6):923–34. doi: 10.1021/acsmedchemlett.2c00052 (PMC9190043; doi:10.1021/acsmedchemlett.2c00052)
Supplement: Supplementary file 1 — ml2c00052_si_001.pdf [file ml2c00052_si_001.pdf]

Supporting information

Discovery of a High AffinityAdenosine A_1/A_3 Receptor Antagonist with a Novel 7-Amino-Pyrazolo[3,4-d]pyridazine Scaffold

Anna Suchankova, [‡,x] Margarita Stampelou, [†,x] Klontiana Koutsouki, [†,x,††] Athanasios Pousias, [†] Lakshiv Dhingra,[‡] Kerry Barkan,[‡] Nicole Pouli,[†] Panagiotis Marakos, [†] Roxane Tenta, [††] Antonios Kolocouris, [†,y,*] Nikolaos Lougiakis [†,y, *] Graham Ladds, [‡, y,*]

*Address for correspondence.*Graham Ladds, Email: **grl30@cam.ac.ukz or** Nikolaos Lougiakis, Email: **nlougiak@pharm.uoa.gr or** Antonios Kolocouris,Email: **ankol@pharm.uoa.gr**

Supplementary Scheme 1

Supplementary Figures 1 – 4

Supplementary Tables 2

Supplementary Methods

Number of pages: 32

Residues	3.33	5.29	5.30	5.38	5.42	6.48	6.51	6.52	6.55	6.66	6.67	7.32	7.35	7.36	7.39
A_1R	L	F	E	M	N	W	L	H	N	K	K	S	T	Y	I
A_3R	L	F	V	M	S	W	L	S	N	V	V	Q	L	Y	I
$A_{2B}R$	L	F	V	M	N	W	V	H	N	K	N	K	M	N	I

Scheme S1.Sequence alignment of the residues surrounding the binding site of A_1R, A_3R,$A_{2B}R$ (Ballesteros-Weinstein[1] numbering is applied). Colored in yellow columns show residues that differ inside chain polarity, volume, rigidity.

Table S1. Chemical structures, antagonistic potencies (pEC_{50} in presence of NECA[a]) of 7-amino-pyrazolo[3,4-*d*]pyridazines **10a-c, 15a-c** against $A_{2A}R$ and $A_{2B}R$.

	$A_{2A}R$ (10 µM)	$A_{2B}R$ (10 µM)	
Compound	pEC_{50} of NECA in presence of compound[a]	pEC_{50} of NECA in presence of compound[a]	pK_d[b]
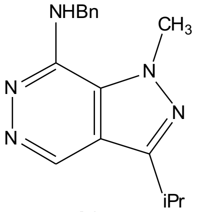 **10a**	5.64 ± 0.38	7.01 ± 0.10	4.79± 0.15
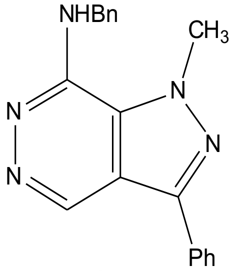 **10b**	5.82 ± 0.33	6.39 ± 0.07*	5.76± 0.14
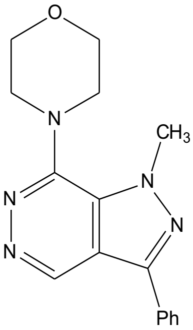 **10c**	5.60 ± 0.52	7.23 ± 0.10	N.B.
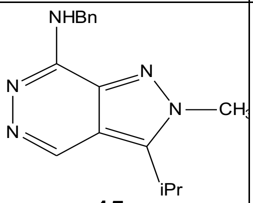 **15a**	6.17 ± 0.47	6.87 ± 0.09	5.09± 0.15
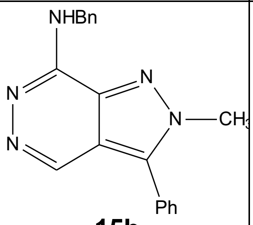 **15b**	5.71 ± 0.46	7.22 ± 0.13	N.B.

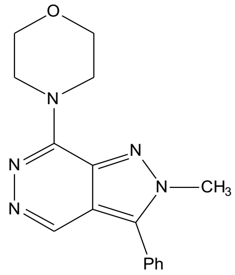 **15c**	5.84 ± 0.46	7.24 ± 0.10	N.B.
vehicle	6.42 ± 0.17	7.22 ± 0.12	-

[a]Mean ± SEM; Functional activities (pEC_{50} values of NECA in the presence ofeither 1 μM ligands or vehicle) as mean ± standard error of the mean (SEM) of at least 3 independent repeats, conducted in duplicate Statistical significance compared to NECA was determined, at $p < 0.05$, through One-Way ANOVA with Dunnett's post-test (*, $p < 0.05$).

[b]Mean ± SEM; Equilibrium binding affinities of the ligands measured with NanoBRET against WT $A_{2B}R$; NECA was used as positive control as described in ref.[2]in the main manuscript

Supplementary MD simulations and MM-GBSA calculations results

Representative MD simulations results for **10b** and **15b** against A_1R**,** A_3R and $A_{2B}R$ and for **10a**, **10c** against A_1R are included (Table S2).

The MM-GBSA [2-4]method with the OPLS2005 [5,6] force field and using a hydrophobic slab [7,8] as implicit membrane model and including waters in the orthosteric binding area, in a radius of 4 Å from the center of mass of the ligand provides a fair ranking for the binding of ligand **10b** against the three receptors A_1R, A_3R or $A_{2B}R$ and for ligands binding **10a-10c** inside A_1R but did not differentiate **10b** and **15b** inside A_1R (Table S2). This is due the lack of accuracy of the MM-GBSA [3-4] method.

Table S2. MD simulations results of 7-amino-pyrazolo[3,4-d]pyridazines **10a-c**, **15b** in complex with A_1R, A_3R or $A_{2B}R$ and MM-GBSA calculations results for **10a-c** in complex with A_1R.

Compound	A_1R			A_3R			$A_{2B}R$		
	$RMSD_{lig}$ [a]	RMSD protein [b]	ΔG_{eff} [c]	$RMSD_{lig}$ [a]	RMSD protein [b]	ΔG_{eff} [c]	$RMSD_{lig}$ [a]	RMSD protein [b]	ΔG_{eff} [c]
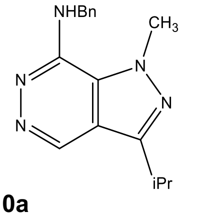 **10a**	2.21 ± 0.42	2.14 ± 0.14	-94.50 ± 5.93	-	-	-	-	-	-
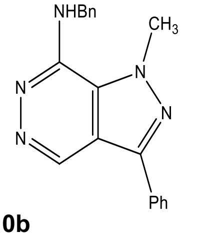 **10b**	1.29 ± 0.31	1.21 ± 0.17	-96.42 ± 5.93	4.88 ± 0.66	2.01 ± 0.21	-101.98 ± 5.02	1.53 ± 0.41	2.08 ± 0.56	-94.19 ± 6.06
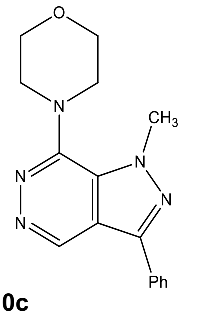 **10c**	1.53 ± 0.37	1.66 ± 0.24	-85.35 ± 5.97	-	-	-	-	-	-
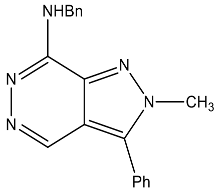 **15b**	2.38 ± 0.45	2.03± 0.19	-	3.19 ± 0.45	2.86 ± 0.15	-	6.77 ± 0.38	2.50 ± 0.14	-

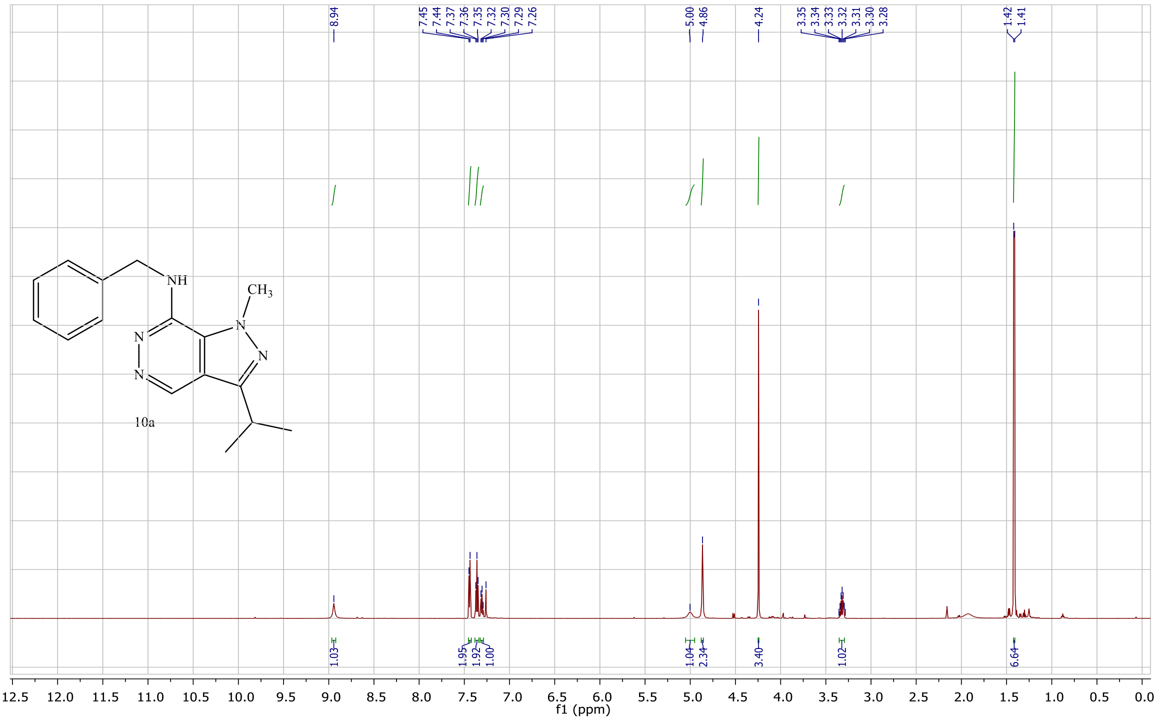
8.94
7.45 7.44 7.37 7.36 7.35 7.32 7.30 7.29 7.26
5.00 4.86
4.24
3.35 3.34 3.33 3.32 3.31 3.30 3.28
1.42 1.41
1.03
1.95 1.92 1.00
1.04 2.34
3.40
1.02
6.64
NH
CH3
N
N
N
N
10a
12.5 12.0 11.5 11.0 10.5 10.0 9.5 9.0 8.5 8.0 7.5 7.0 6.5 6.0 5.5 5.0 4.5 4.0 3.5 3.0 2.5 2.0 1.5 1.0 0.5 0.0
f1 (ppm)

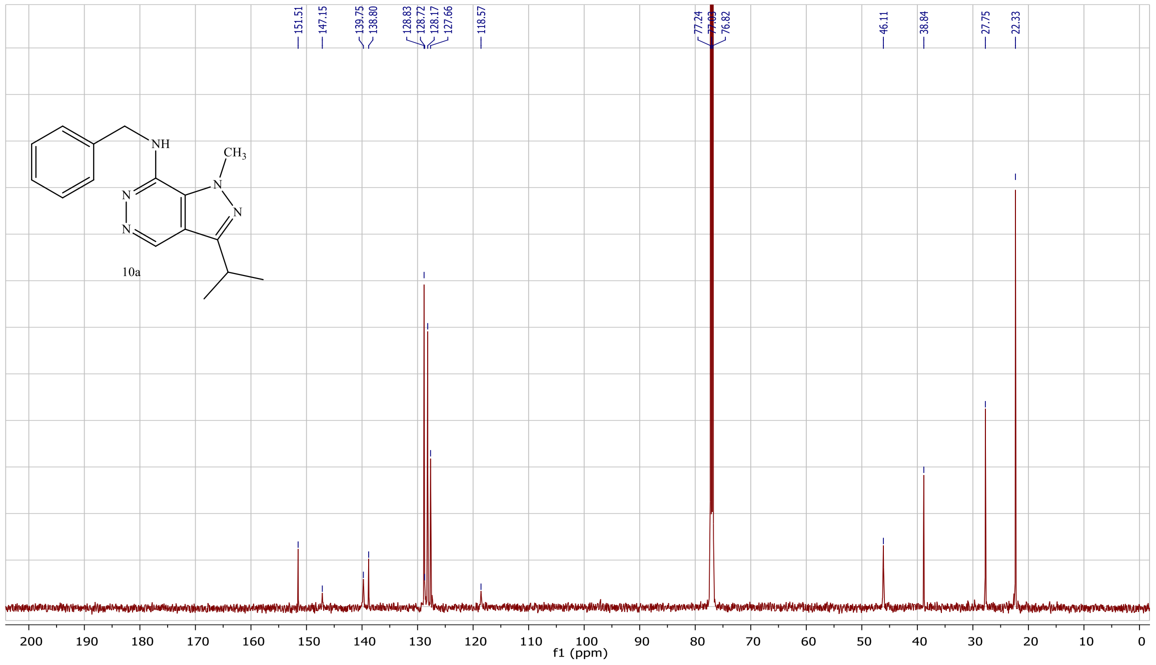
151.51
147.15
139.75 138.80
128.83 128.72 128.17 127.66
118.57
77.24 76.82
46.11
38.84
27.75
22.33
NH
CH3
N
N
N
N
10a
200 190 180 170 160 150 140 130 120 110 100 90 80 70 60 50 40 30 20 10 0
f1 (ppm)

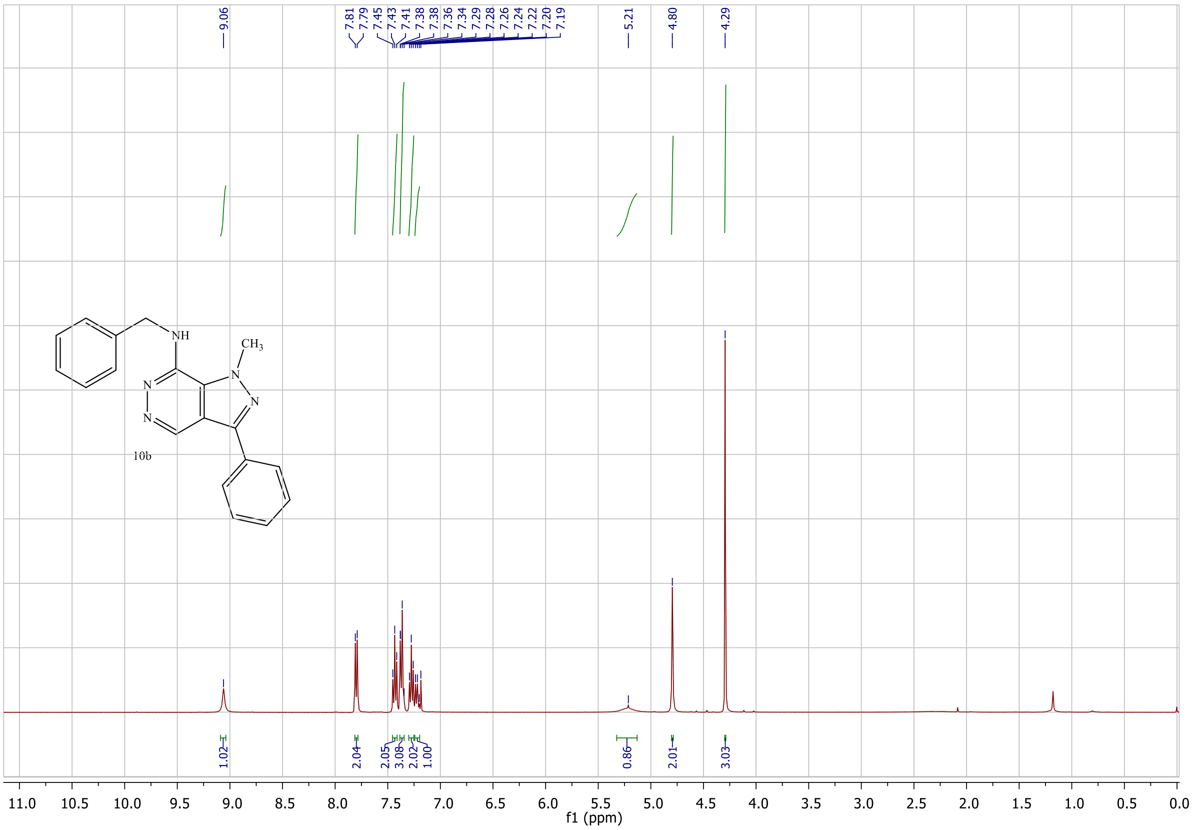
9.06
7.81 7.79 7.45 7.43 7.41 7.38 7.38 7.36 7.34 7.29 7.28 7.26 7.24 7.22 7.20 7.19
5.21
4.80
4.29
NH
CH3
N
10b
1.02
2.04
2.05
3.08
2.02
1.00
0.86
2.01
3.03
f1 (ppm)

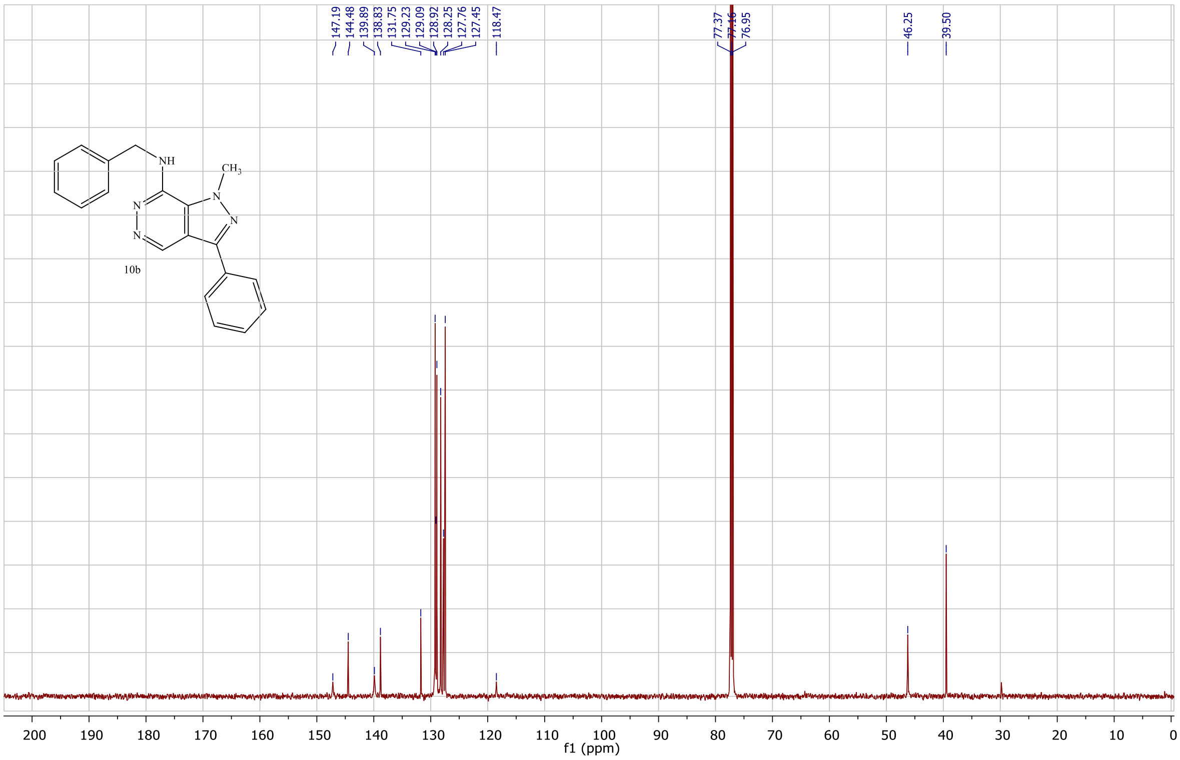
147.19 144.48 139.89 138.83 131.75 129.23 129.09 128.92 128.25 127.76 127.45
118.47
77.37 77.16 76.95
46.25
39.50
NH
CH3
N
10b
f1 (ppm)

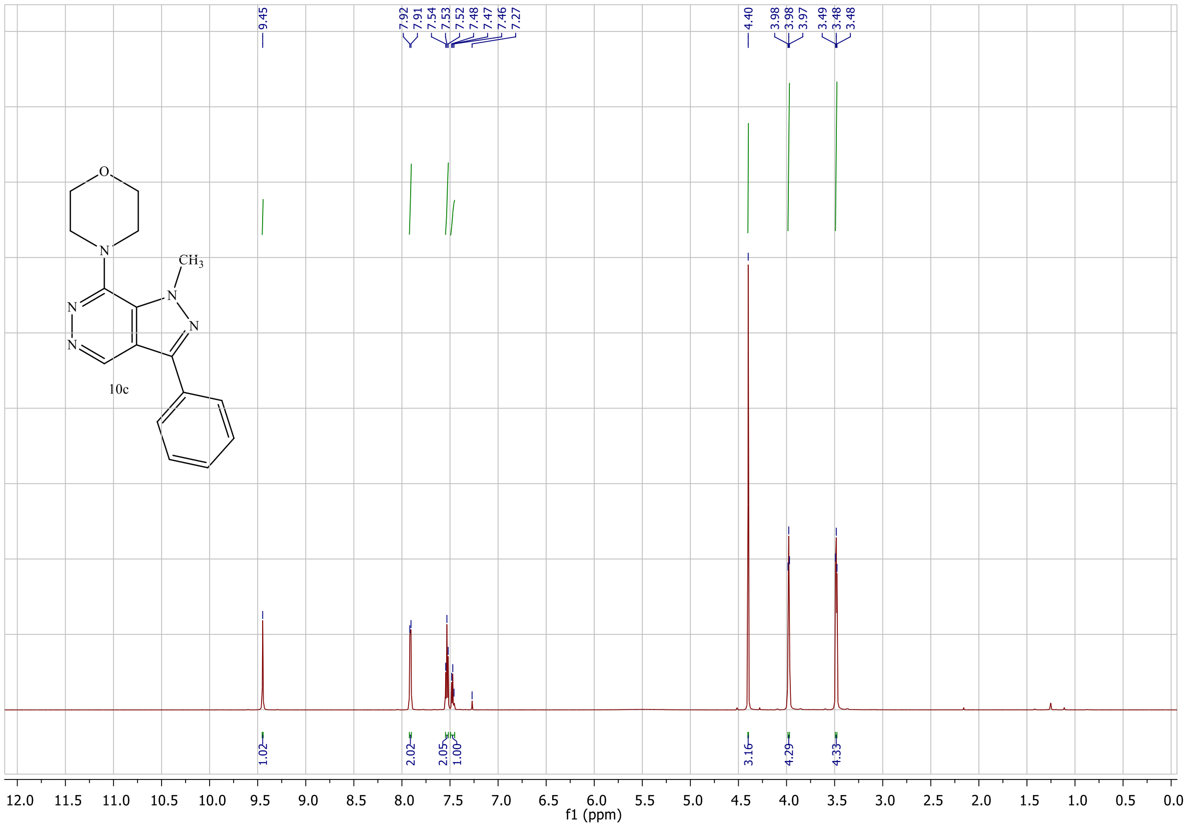

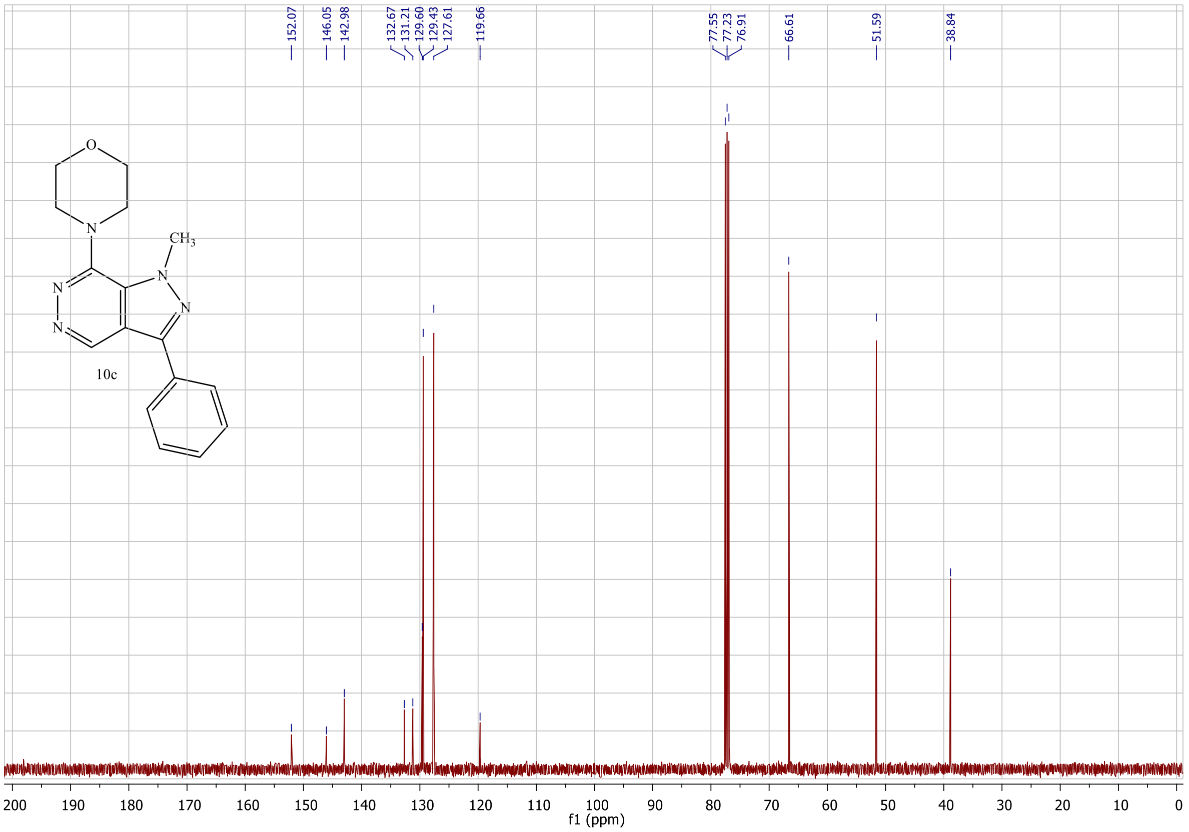

Figure S1. ^{1}H and ^{13}C NMR spectra of compounds 10a-**c** in $CDCl_3$.

A

$A_{2B}R$ - 10b

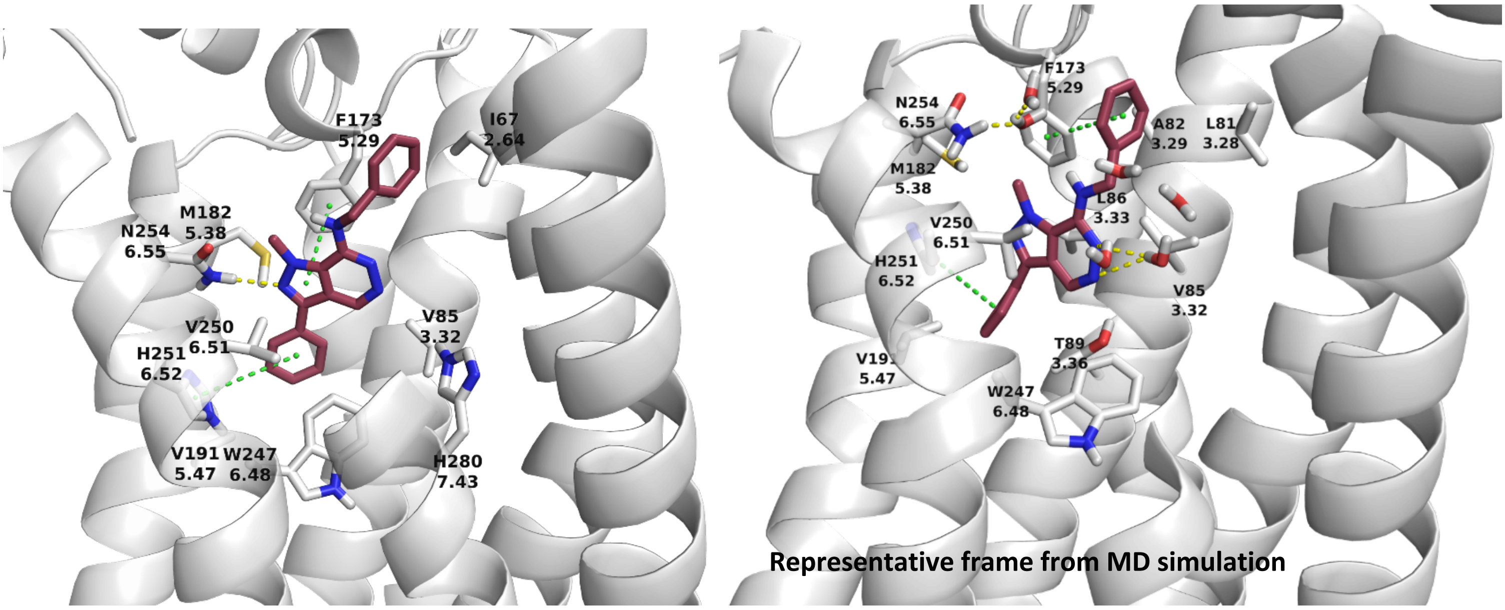

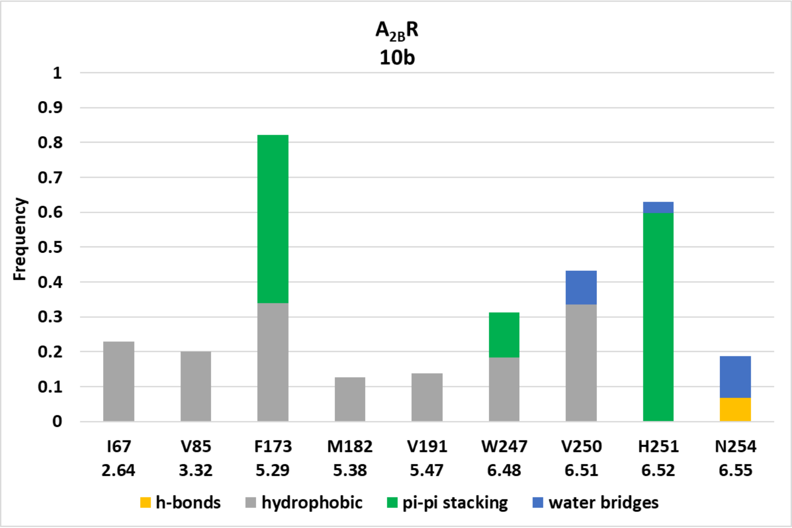

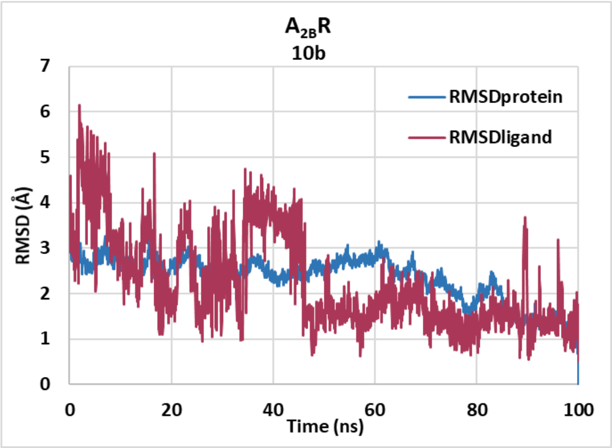

The mean frame from MD simulations was close to the starting docking pose in $A_{2B}R$ ($RMSD_{lig}$= 1.53 Å). The interaction profile for **10b** was very similar inside $A_{2B}R$ to that observed for the A_1R (Figure S2).

B

A_3R - 10b

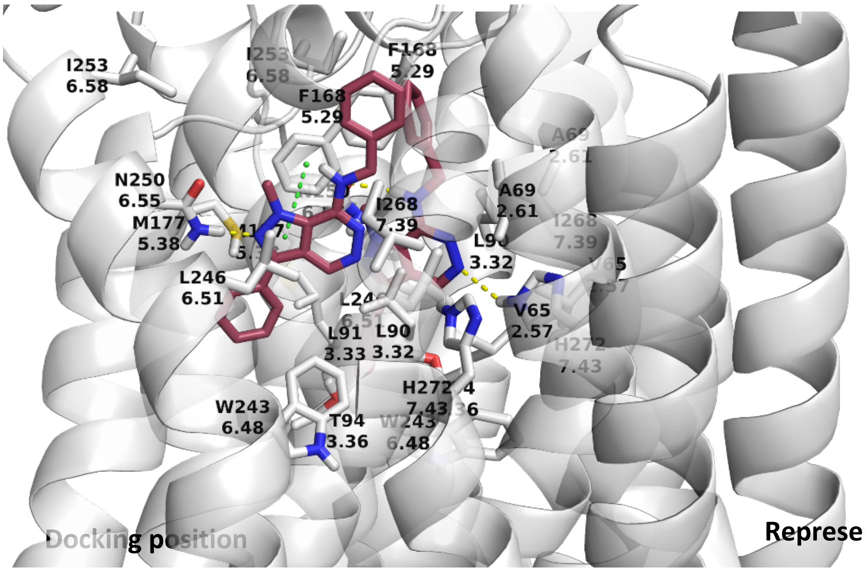

Representative frame from MD simulation

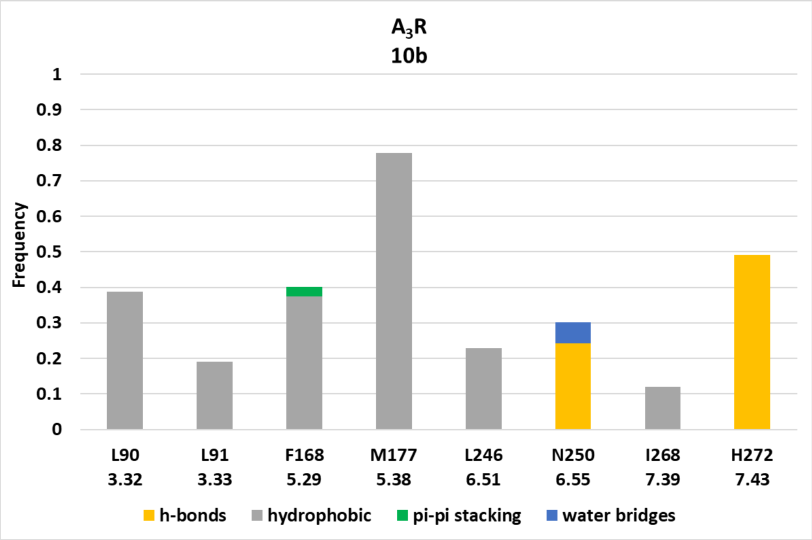

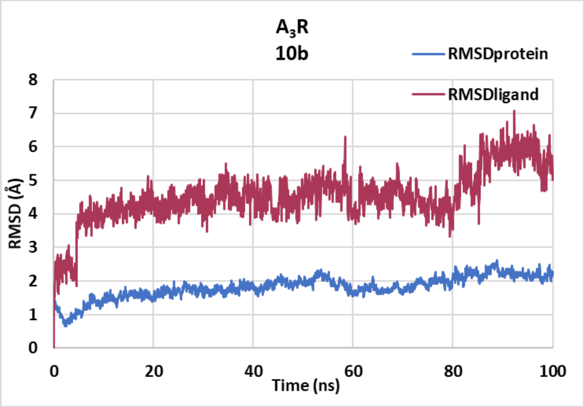

C

A_{2B}R - 15b

For **15b** – A_{2B}R complex a similar binding pose as **10b** – A_{2B}R was used. After 100ns molecular dynamic simulation, **15b** leaves the binding pocket and enters the membrane area through an opening between TM6 and TM7. The RMSD of this ligand (6.77 ± 0.38 Å) is also indicative of a ligand translation from the starting position.

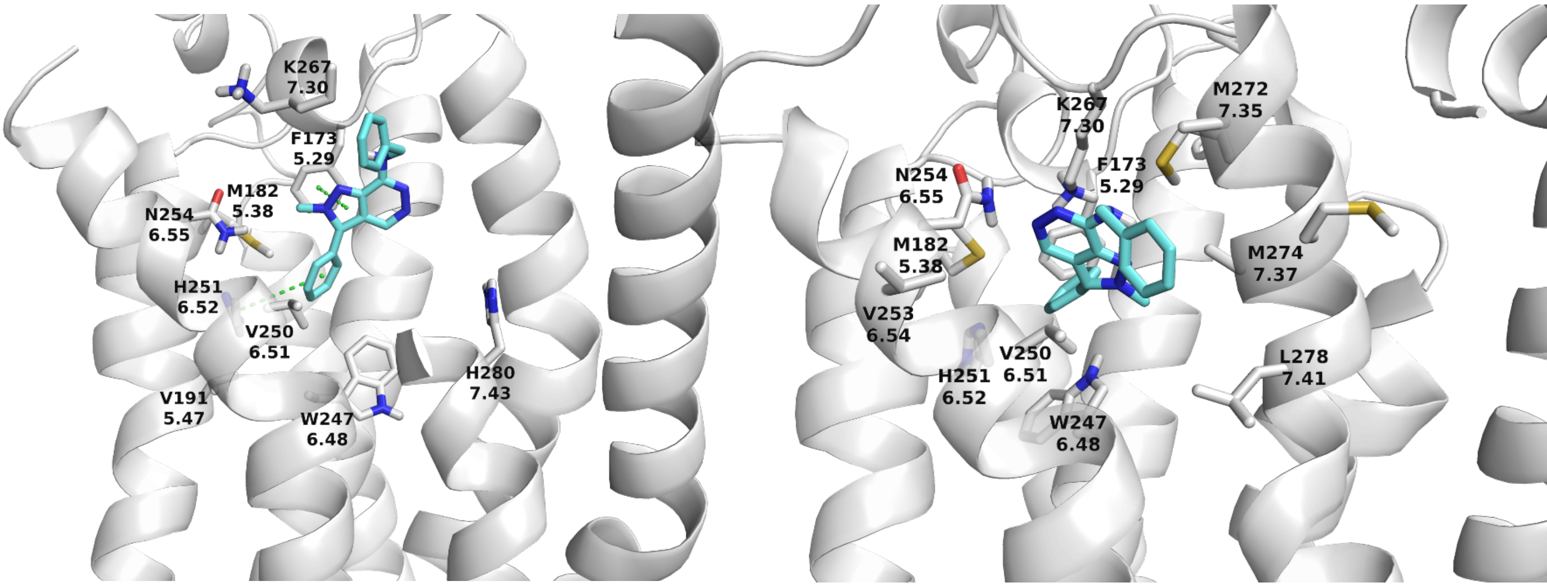

Representative frame from MD simulation

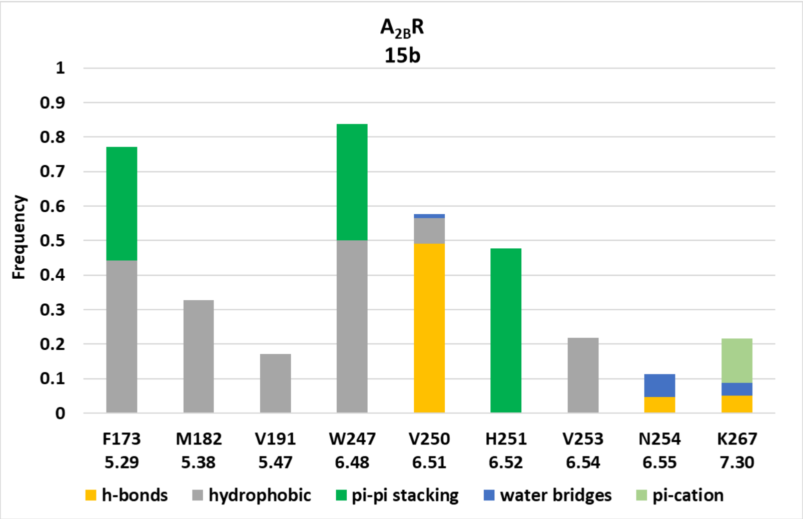

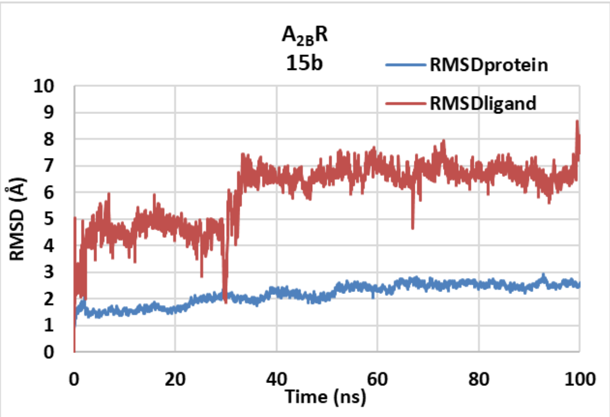

D

A_3R 15b

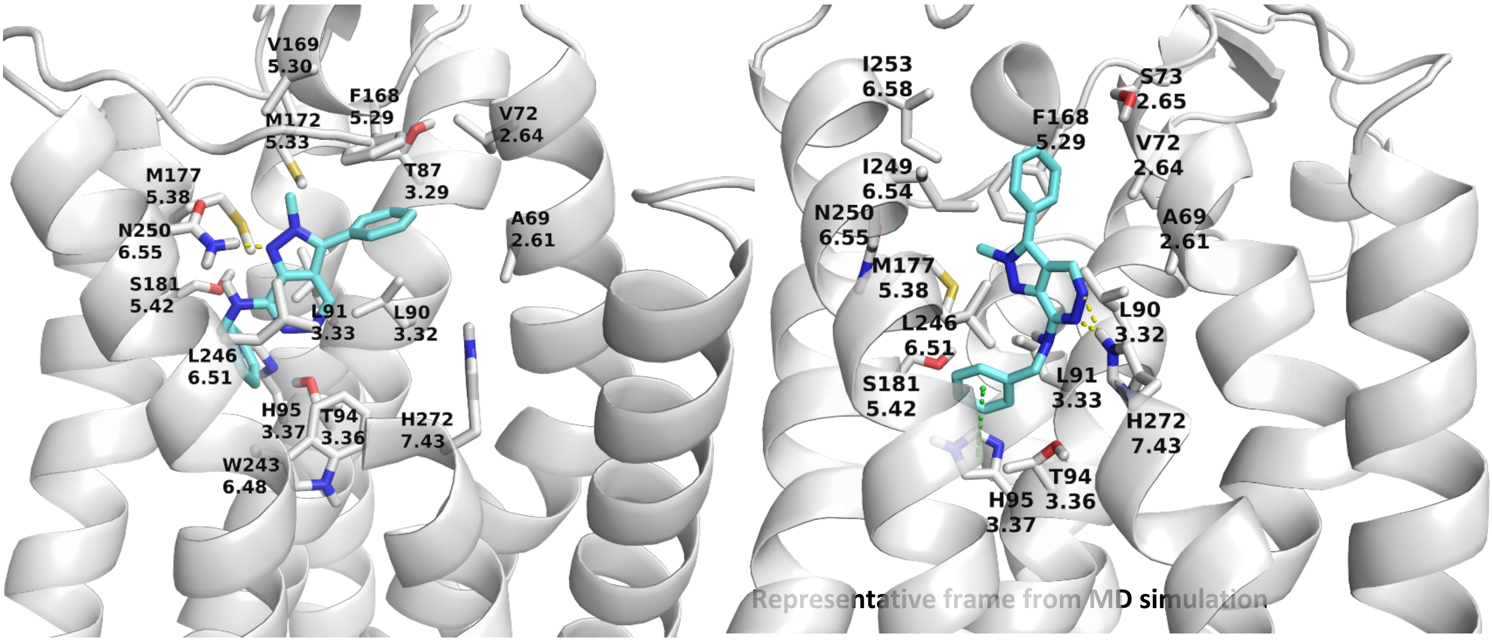

Figure S2. Docking poses and representative frames, receptor-ligand interaction frequency histograms and RMSD graphs of protein Ca ($RMSD_{protein}$;blue plots) and ligand heavy atoms ($RMSD_{ligand}$; red plots) from 100ns-MD simulations of **10b**(**A**)-(**B**) inside the orthosteric binding area of WT A_3R or $A_{2B}R$ and (**C**),(**D**) **15b** inside the orthosteric binding area of WT $A_{2B}R$,A_3R, respectively. Bars are plotted only for residues with interaction

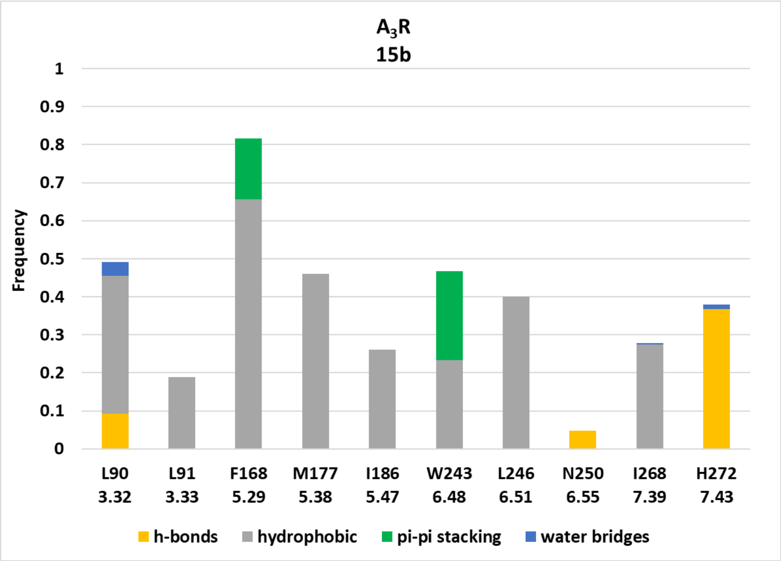

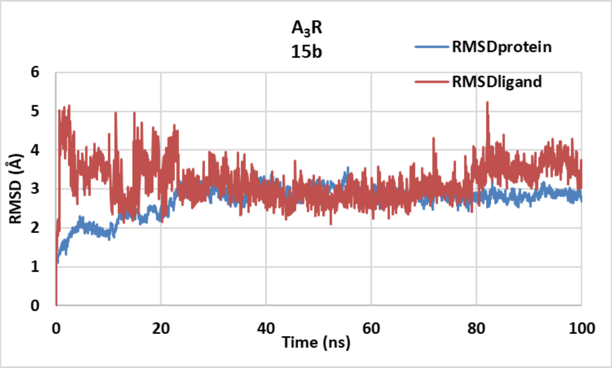

frequencies ≥ 0.2. Color scheme: Ligand=cyan sticks, receptor=white cartoon and sticks, hydrogen bonding interactions=yellow (dashes or bars), π-π interactions=green (dashes or bars); hydrophobic interactions=grey; water bridges=blue. For the protein models of A_3R, $A_{2B}R$ were used the homology model derived from $A_{2A}R$ (PDB ID 3EML) [4]in complex with a highest-scored docking pose of tested compound.

A similar heterocyclic system with our compounds found in the literature is the 4-(2-phenethyl)amino 1-phenylethyl pyrazolo[3,4-*b*]pyridine which was found selective against A_1R. As has already been described in the literature[5] the docking calculations showed that the 1*H*-pyrazolo[3,4-*b*]pyridine central scaffold has a π–π interaction with F171, a lipophilic interaction with V87 and L250, and 7-*N* forms a hydrogen bond with N254. The 4-(2-phenethyl)amino substituent is oriented towards the extracellular part of the receptor and interacted with I274 while 1-phenylethyl towards the bottom of the receptor.

The representative ligand N9-methyl,N6-benzyl adenine binds A_1R[6]. Our docking calculations show that the adenine ring has a π–π interaction with F171, theimidazole nitrogen forms a hydrogen bond with N254 and thatN6-benzyl is oriented towards the extracellular part of the receptor (Figure S3). We did not find in the literature any information for the activity of N7-methyl, N6-benzyl adenine against ARs; this compound had been developed against CDK kinases.[7]

N9-methyl,N6-benzyl adenine to A_1R

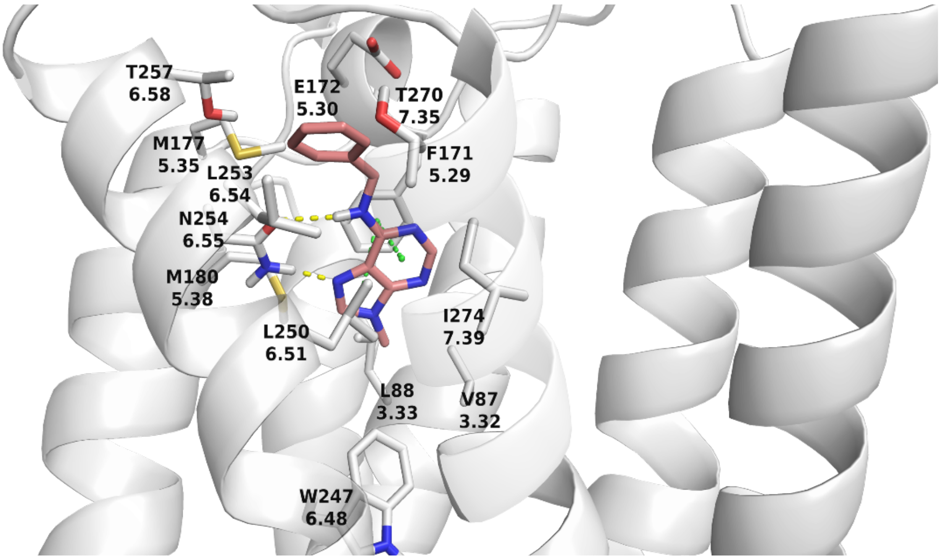

Figure S3. N9-methyl,N6-benzyl adenine inside the orthosteric binding area of WT A_1R; from docking calculations. Color scheme: Ligand=light pink sticks, receptor=white cartoon and sticks, hydrogen bonding interactions=yellow (dashes), π-π interactions=green (dashe). For the protein models of A_1R the experimental structure of the inactive form for A_1R in complex with an antagonist (PDB ID 5UEN [8]) was used for homology modelling.

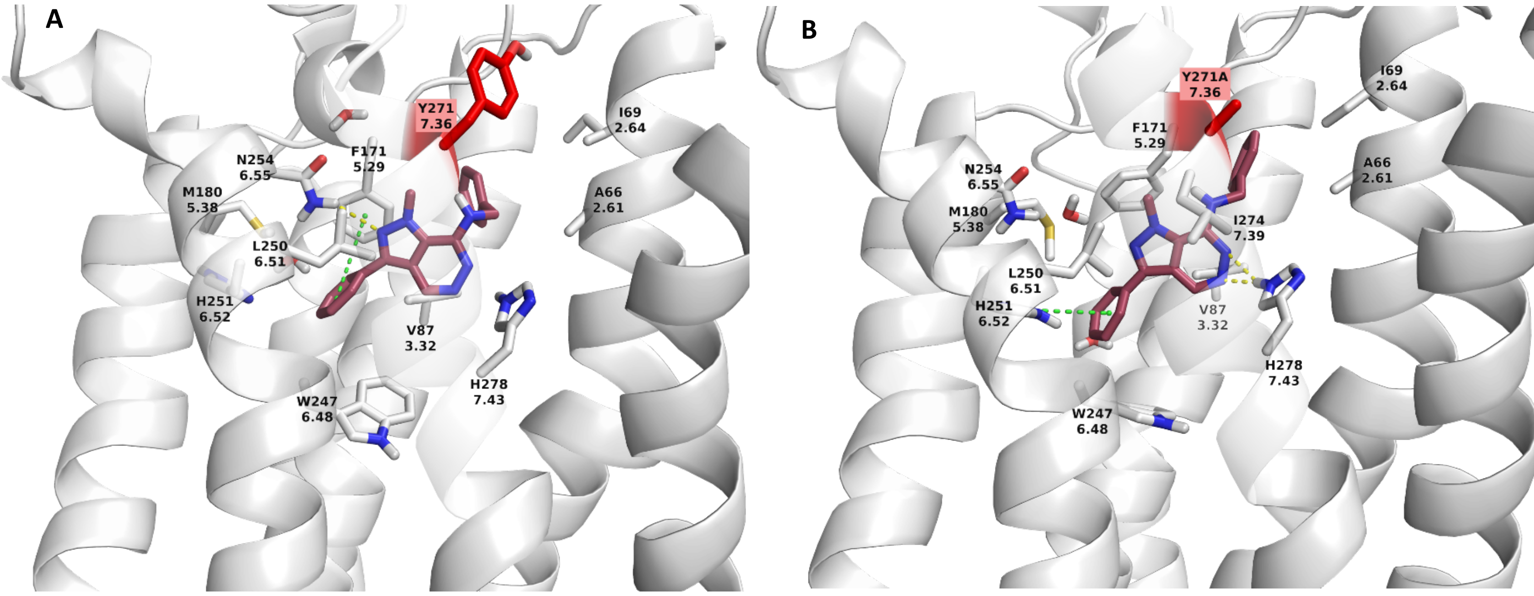
A
Y271
7.36
I69
2.64
N254
6.55
F171
5.29
M180
5.38
A66
2.61
L250
6.51
H251
6.52
V87
3.32
H278
7.43
W247
6.48
B
Y271A
7.36
I69
2.64
F171
5.29
N254
6.55
A66
2.61
M180
5.38
I274
7.39
L250
6.51
H251
6.52
V87
3.32
H278
7.43
W247
6.48

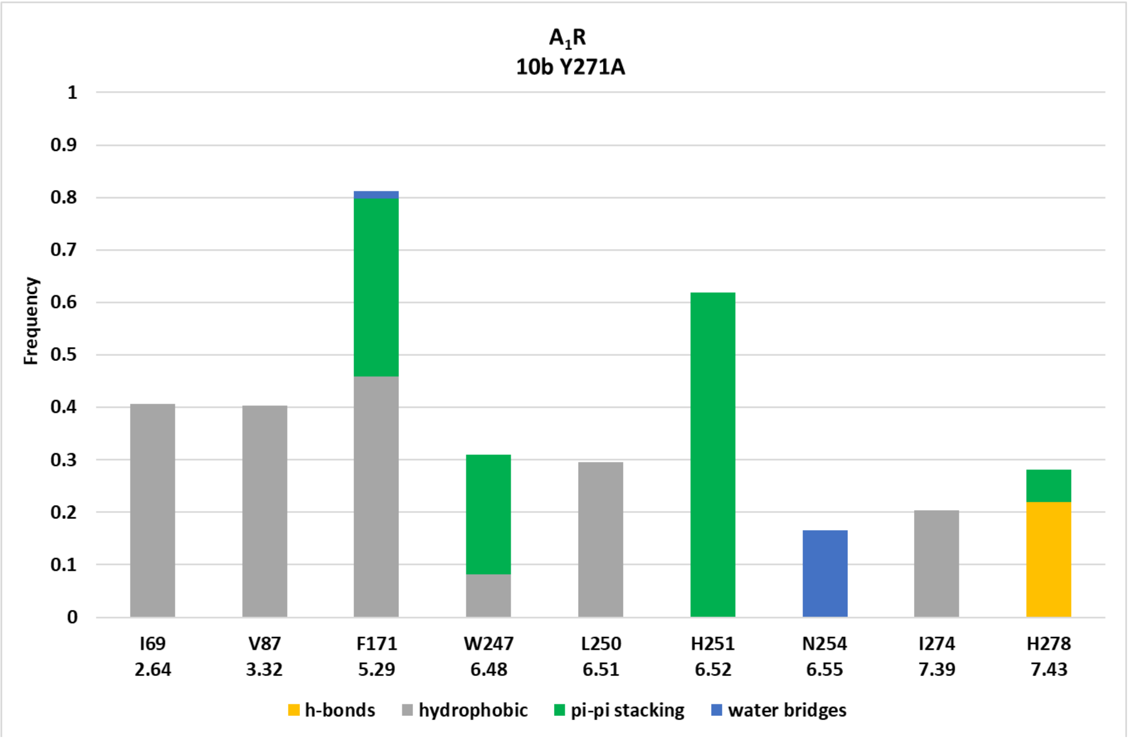
A_1R
10b Y271A
Frequency
1
0.9
0.8
0.7
0.6
0.5
0.4
0.3
0.2
0.1
0
I69 2.64
V87 3.32
F171 5.29
W247 6.48
L250 6.51
H251 6.52
N254 6.55
I274 7.39
H278 7.43
h-bonds
hydrophobic
pi-pi stacking
water bridges

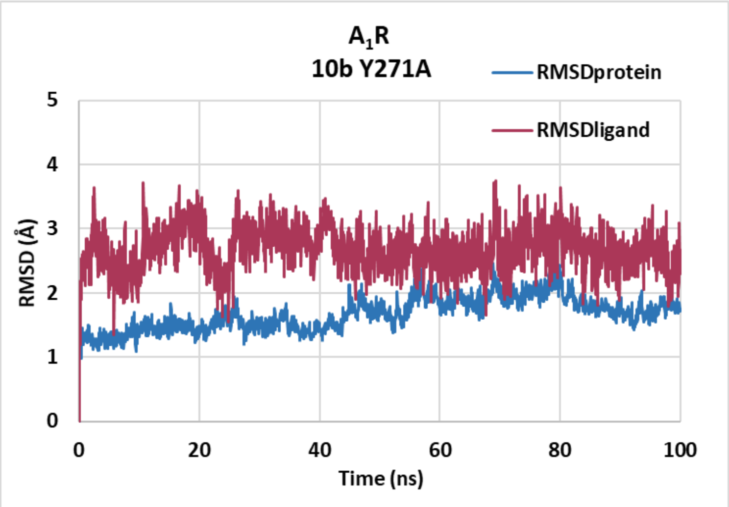

**protein RMSD**	1.87 ± 0.18
**ligand RMSD**	2.65 ± 0.32

Figure S4. Representative frames from 100ns-MD simulations of (A) 10b inside the orthosteric binding area of WT A_1R; (B) **10b** inside mutant Y271A A_1R. Receptor-ligand interaction frequency histogram and RMSD graphs of protein Ca (RMSDprotein; red plots) and ligand heavy atoms (RMSDligand; blue plots). Bars are plotted only for residues with interaction frequencies ≥ 0.2. Color scheme: Ligand=brown sticks, receptor=white cartoon and sticks, hydrogen bonding interactions=yellow (dashes or bars), π-π interactions=green (dashes or bars); hydrophobic interactions=grey; water bridges=blue. For the protein models of A_1R the experimental structure of the inactive form for A_1R in complex with an antagonist (PDB ID 5UEN [8]) was used for homology modelling.

A small TM2 curvature already exists in the model of A_3R generated using as template the crystallographic structure of $A_{2A}R$ (PDB ID 3EML)[8].

Methods

Safety

No unexpected or unusually high safety hazards were encountered during the proceeding of this project.

Chemistry

Melting points were determined on a Büchi apparatus and are uncorrected. ^1H NMR spectra and 2D spectra were recorded on a Bruker Avance III 600 or a Bruker Avance DRX 400 instrument, whereas ^{13}C NMR spectra were recorded on a Bruker Avance III 600 or a Bruker AC 200 spectrometer in deuterated solvents and were referenced to TMS (δ scale). The signals of ^1H and ^{13}C spectra were unambiguously assigned by using the 2D NMR spectra COSY, NOESY, HMQC, and HMBC. Mass spectra were recorded with a LTQ Orbitrap Discovery instrument, possessing an Ionmax ionization source. The purity of all the target compounds was >95% as ascertained by ^1H-NMR and elemental analysis. Elemental analyses were undertaken using a PerkinElmer PE 240C elemental analyzer and the measured values for C, H, and N were within ±0.4% of the theoretical values. Flash

chromatography was performed on Merck silica gel 60 (0.035 - 0.070 mm). Analytical thin layer chromatography (TLC) was carried out on precoated (0.25 mm) silica gel F-254 plates.

General procedure for the synthesis of derivatives 4a-5a and 4b-5b

Sodium hydride (1.30 g, 32.4 mmol, 60% dispersion in mineral oil) was added into a solution of pyrazole **3a** or **3b** (27.0 mmol) in anhydrous DMF (25 mL) at 0 °C, under argon, and this solution was stirred for 15 min, followed by dropwise addition of iodomethane (2.5 mL, 32.4 mmol). This reaction mixture was stirred at r.t for 1 h. Upon completion of the reaction, the solvent was evaporated under reduced pressure and the residue was extracted with dichloromethane (3 x 150 mL) and water (150 mL). The combined organic layers were dried over Na_2SO_4 and evaporated under reduced pressure. The crude product was then purified by column chromatography (silica gel) using a mixture of cyclohexane/ethyl acetate 10/1-10/2 as the eluent to provide pure compounds **4a**–**5a** and **4b**–**5b.**

Ethyl 3-isopropyl-1-methyl-1H-pyrazole-5-carboxylate (**4a**): Yield 28%. Colorless oil. ^{1}H- and ^{13}C-NMR spectra identical to those referred in bibliography.[11]

Ethyl *5-isopropyl-1-methyl-1H-pyrazole-3*-carboxylate (***5a***)*:* Yield 72%. Pale yellow oil.^{1}H-NMR (600 MHz, $CDCl_3$) δ (ppm) 6.63 (s, 1H, H-4), 4.31 (q, J = 7.1 Hz, 2H, CH_2CH_3), 4.10 (s, 3H, NCH_3), 2.95 (hept, J = 7.0 Hz, 1H, $CH(CH_3)_2$), 1.36 (t, J = 7.1 Hz, 3H, CH_2CH_3), 1.24 (d, J = 7.0 Hz, 6H, $CH(CH_3)_2$). ^{13}C-NMR (151 MHz, $CDCl_3$) δ (ppm) 159.86 (C=O), 157.29 (C-3), 132.58 (C-5), 107.41 (C-4), 60.59 (CH_2CH_3), 38.93 (NCH_3), 27.52 ($CH(CH_3)_2$), 22.61 ($CH(CH_3)_2$), 14.12 (CH_2CH_3). HRMS (ESI) m/z: Calcd for $C_{10}H_{17}N_2O_2$: $[M+H]^+$ =197.1285, found 197.1281.

Ethyl 1-methyl-3-phenyl-1H-pyrazole-5-carboxylate (**4b**): Yield 63%. Pale yellow oil. ^{1}H- and ^{13}C-NMR spectra identical to those referred in bibliograph. [12]

Ethyl 1-methyl-5-phenyl-1H-pyrazole-3-carboxylate (**5b**): Yield 32%. Pale yellow oil. ^{1}H- and ^{13}C-NMR spectra identical to those referred in bibliography. [12]

Ethyl 4-bromomethyl-3-isopropyl-1-methyl-1*H*-pyrazole-5-carboxylate (**6a**): In a 33% HBr solution in acetic acid (3mL) were added the carboxylate **4a** (0.2 g, 1.0 mmol) and paraformaldehyde (98 mg, 3.3 mmol) and the resulting mixture was heated at 90 °C for 3.5 h. The mixture was then poured into ice, made alkaline using $NaHCO_3$ (pH~8) and extracted with ethyl acetate (3x30 mL). The organic solvent was dried (Na_2SO_4) and evaporated to dryness and the residue was purified by column chromatography (silica gel) using a mixture of dichloromethane/ethyl acetate 10/0.3-10/1 as the eluent to provide 0.1 g (33 %) of pure compound 6a as an oil. ^{1}H-NMR (600 MHz, $CDCl_3$) δ (ppm) 4.67 (s, 2H, CH_2Br), 4.40 (q, J = 7.1 Hz, 2H, CH_2CH_3), 4.09 (s, 3H, NCH_3), 3.04 (hept, J = 7.0 Hz, 1H, $CH(CH_3)_2$), 1.43 (t, J = 7.1 Hz, 3H, CH_2CH_3), 1.30 (d, J = 7.0 Hz, 6H, $CH(CH_3)_2$). ^{13}C-NMR (151 MHz, $CDCl_3$) δ (ppm) 159.87 (C=O), 156.17 (C-3), 130.48 (C-5), 119.21 (C-4), 61.41 (CH_2CH_3), 40.10 (NCH_3), 26.08 ($CH(CH_3)_2$), 23.14 ($CH(CH_3)_2$), 22.34 (CH_2Br), 14.26 (CH_2CH_3). HRMS (ESI) m/z: Calcd for $C_{11}H_{18}BrN_2O_2$: $[M+H]^+$ =289.0547, found 289.0542.

Ethyl 4-bromomethyl-1-methyl-3-phenyl-1*H*-pyrazole-5-carboxylate (**6b**): This compound was prepared following a method analogous to that of **6a**, starting from **4b**. Yield 72%. Mp: 47-49 °C (pentane). ^{1}H-NMR (600 MHz, $CDCl_3$) δ (ppm) 7.78 (d, J = 7.4 Hz, 2H, H-2’, H-6’), 7.50 (t, J = 7.4 Hz, 2H, H-3’, H-5’), 7.43 (t, J = 7.4 Hz, 1H, H-4’), 4.76 (s, 2H, CH_2Br), 4.49 (q, J = 7.1 Hz, 2H, $C\underline{H_2}CH_3$), 4.24 (s, 3H, NCH_3), 1.51 (t, J = 7.1 Hz, 3H, $CH_2C\underline{H_3}$). ^{13}C-NMR (151 MHz, $CDCl_3$) δ (ppm) 159.64 (C=O), 149.96 (C-3), 131.91 (C-1’), 131.55 (C-5), 128.84 (C-3’, C-5’), 128.49 (C-4’), 128.21 (C-2’, C-6’), 120.02 (C-4), 61.65 ($\underline{C}H_2CH_3$), 40.51 (NCH_3), 24.55 (CH_2Br), 14.22 ($CH_2\underline{C}H_3$). HRMS (ESI) m/z: Calcd for $C_{14}H_{16}BrN_2O_2$: $[M+H]^+$ =323.0390, found 323.0388.

Ethyl 4-formyl-3-isopropyl-1-methyl-1*H*-pyrazole-5-carboxylate (**7a): ** To a solution of the bromide **6a** (150 mg, 0.5 mmol) in dry acetonitrile (2 mL) was added under argon N-methylmorpholine-N-oxide (120 mg, 1.0 mmol) and the mixture was stirred for 24 h. The solvent was then vacuum-evaporated, water was added to the residue, and it was extracted with ethyl acetate. The organic phase was dried (Na_2SO_4) and concentrated to dryness to provide in high purity compound 7a (100 mg, 86 %) as an oil. ^{1}H-NMR (400 MHz, $CDCl_3$) δ (ppm) 10.32 (s, 1H, CHO), 4.40 (q, J = 7.1 Hz, 2H, $C\underline{H_2}CH_3$), 4.07 (s, 3H, NCH_3), 3.47 (hept, J = 7.0 Hz, 1H, $C\underline{H}(CH_3)_2$), 1.37 (t, J = 7.1 Hz, 3H, $CH_2C\underline{H_3}$), 1.20 (d, J = 7.0 Hz, 6H, $CH(C\underline{H_3})_2$). ^{13}C-NMR (50 MHz, $CDCl_3$) δ (ppm) 187.34 (CHO), 159.19 (C=O), 158.82 (C-3), 135.57 (C-5), 120.36 (C-4), 62.18 ($\underline{C}H_2CH_3$), 39.89 (NCH_3), 27.06 ($\underline{C}H(CH_3)_2$), 21.42 ($CH(\underline{C}H_3)_2$), 14.13 ($CH_2\underline{C}H_3$). HRMS (ESI) m/z: Calcd for $C_{11}H_{17}N_2O_3$: $[M+H]^+$ =225.1234, found 225.1227.

Ethyl 4-formyl-1-methyl-3-phenyl-1H-pyrazole-5-carboxylate (**7b**):This compound was prepared following a method analogous to that of **7a**, starting from **6b**. Yield 85%. Mp: 73-75 °C (CH_2Cl_2-pentane). ^{1}H-NMR (400 MHz, $CDCl_3$) δ (ppm) 10.37 (s, 1H, CHO), 7.73 (d, J = 7.4 Hz, 2H, H-2’, H-6’), 7.47 – 7.36 (m, 3H, H-3’, H-5’, H-4’), 4.47 (q, J = 7.1 Hz, 2H, $C\underline{H_2}CH_3$), 4.19 (s, 3H, NCH_3), 1.43 (t, J = 7.1 Hz, 3H, $CH_2C\underline{H_3}$). ^{13}C-NMR (50 MHz, $CDCl_3$) δ (ppm) 186.09 (CHO), 159.14 (C=O), 151.67 (C-3), 136.31 (C-5), 131.36 (C-1’), 129.16 (C-2’, C-6’), 129.05 (C-4’), 128.12 (C-3’, C-5’), 120.83 (C-4), 62.49 ($\underline{C}H_2CH_3$), 40.14 (NCH_3), 14.13 ($CH_2\underline{C}H_3$). HRMS (ESI) m/z: Calcd for $C_{14}H_{15}N_2O_3$: $[M+H]^+$ =259.1078, found 259.1077.

3-Isopropyl-1-methyl-1,6-dihydro-7H-pyrazolo[3,4-d]pyridazin-7-one (**8a**):To a solution of the carboxylate **7a** (0.11 g, 0.49 mmol) in ethanol (2.5 mL) was added dropwise hydrazine (20 μL, 0.49 mmol, 80 % in water) and a few drops of HCl 36 % and the mixture was heated at 90 °C for 1 h. The solvent was vacuum-evaporated, water was added to the residue and it was extracted with ethyl acetate (3x20 mL). The organic extracts were dried (Na_2SO_4) and evaporated to dryness and the residue was purified by column chromatography (silica gel) using a mixture of cyclohexane/ethyl acetate 8/2-6/4 as the eluent, to result in 60 mg (67 %) of pure compound **8a**. M.p.: 178-180 °C (EtOAc). ^{1}H-NMR (400 MHz, DMSO-d_6) δ (ppm) 12.63 (s, D_2O exchang., 1H, NH), 8.38 (s, 1H, H-4), 4.18 (s, 3H, NCH_3), 3.26 (hept, J = 7.0 Hz, 1H, $C\underline{H}(CH_3)_2$), 1.29 (d, J = 7.0 Hz, 6H, $CH(C\underline{H_3})_2$). ^{13}C-NMR (151 MHz, DMSO-d_6) δ (ppm) 154.40 (C=O), 151.68 (C-3), 133.08 (C-4), 132.83 (C-7a), 116.97 (C-3a), 37.73

(NCH$_3$), 26.84 (CH(CH$_3$)$_2$), 22.27 (CH(CH$_3$)$_2$). HRMS (ESI) m/z: Calcd for $C_9H_{13}N_4O$: $[M+H]^+$ =193.1084, found 193.1079.

1-Methyl-3-phenyl-1,6-dihydro-7H-pyrazolo[3,4-d]pyridazin-7-one (**8b**)*:* This compound was prepared following a method analogous to that of **8a**, starting from **7b**. Yield 82%. Mp: 253-255 °C (EtOH). ^{1}H-NMR (400 MHz, DMSO-d$_6$) δ (ppm) 12.83 (s, D_2O exchang., 1H, NH), 8.62 (s, 1H, H-4), 7.93 (d, J = 7.3 Hz, 2H, H-2', H-6'), 7.52 (t, J = 7.3 Hz, 2H, H-3', H-5'), 7.46 (t, J = 7.3 Hz, 1H, H-4'), 4.33 (s, 3H, NCH$_3$). ^{13}C-NMR (151 MHz, DMSO-d$_6$) δ (ppm) 154.16 (C=O), 144.06 (C-3), 133.65 (C-7a), 133.03 (C-4), 131.22 (C-1'), 129.15 (C-3', C-5'), 128.88 (C-4'), 126.97 (C-2', C-6'), 116.87 (C-3a), 38.31 (NCH$_3$). HRMS (ESI) m/z: Calcd for $C_{12}H_{11}N_4O$: $[M+H]^+$ =227.0928, found 227.0926.

7-Chloro-3-isopropyl-1-methyl-1H-pyrazolo[3,4-d]pyridazine (**9a**)**:**A mixture of compound **8a** (40 mg, 0.2 mmol) in phosphorus oxychloride (2 mL) was heated at 110 °C for 8h. Upon completion of the reaction (determined by TLC), the excess of phosphorus oxychloride was vacuum-evaporated and the synthesized chloride **9a** was used for the next step without further purification due to the relative ease of hydrolysis.

7-Chloro-1-methyl-3-phenyl-1H-pyrazolo[3,4-d]pyridazine (**9b**)**:**This compound was prepared following a method analogous to that of **9a**, starting from **8b**. In this case the reaction was completed in2.5 h and the resulting chloride was again not purified.

N-Benzyl-3-isopropyl-1-methyl-1*H*-pyrazolo[3,4-*d*]pyridazin-7-amine (**10a**): To a solution of the chloropyrazolopyridazine 9a (42 mg, 0.2 mmol) in absolute ethanol (3 mL), was added benzylamine (66 μL, 0.6 mmol) and the mixture was heated at 80 °C for 2h. The solvent was then vacuum-evaporated, water was added to the residue, and it was extracted with dichloromethane (3x20 mL). The organic extracts were dried (Na_2SO_4) and evaporated to dryness and the residue was purified by column chromatography (silica gel) using a mixture of cyclohexane/ethyl acetate 4/6 as the eluent, to result in 23 mg (41 %) of pure compound 10a. M.p.: 137-140 °C (Et_2O). ^{1}H-NMR (600 MHz, $CDCl_3$) δ (ppm) 8.94 (s, 1H, H-4), 7.44 (d, J = 7.4 Hz, 2H, H-2, benzyl H-6), 7.36 (t, J = 7.4 Hz, 2H, benzyl H-3, H-5), 7.30 (t, J = 7.4 Hz, 1H, benzyl H-4), 5.00 (s, 1H, NH), 4.86 (s, 2H, benzyl CH$_2$), 4.24 (s, 3H, NCH$_3$), 3.32 (hept, J = 7.0 Hz, 1H, CH(CH$_3$)$_2$), 1.42 (d, J = 7.0 Hz, 6H, CH(CH$_3$)$_2$). ^{13}C-NMR (151 MHz, $CDCl_3$) δ (ppm) 151.51 (C-3), 147.15 (C-7), 139.75 (C-4), 138.80 (benzyl C-1), 128.83 (benzyl C-3, C-5), 128.72 (C-7a), 128.17 (benzyl C-2, C-6), 127.66 (benzyl C-4), 118.57 (C-3a), 46.11 (benzyl CH$_2$), 38.84 (NCH$_3$), 27.75 (CH(CH$_3$)$_2$), 22.33 (CH(CH$_3$)$_2$). Anal. Calcd for $C_{16}H_{19}N_5$: C, 68.30; H, 6.81; N, 24.89. Found: C, 68.54; H, 6.88; N, 24.55.

N-Benzyl-1-methyl-3-phenyl-1*H*-pyrazolo[3,4-*d*]pyridazin-7-amine (**10b**): This compound was prepared following a method analogous to that of **10a**, starting from **9b**. Yield 33%. Mp: 208-210 °C (CH_2Cl_2-Et_2O). ^{1}H-NMR (600 MHz, $CDCl_3$) δ (ppm) 9.06 (s, 1H, H-4), 7.80 (d, J = 7.6 Hz, 2H, H-2', H-6'), 7.43 (t, J = 7.6 Hz, 2H, H-3', H-5'), 7.36 (m, 3H, benzyl H-2, H-6, H-4), 7.28 (t, J = 7.8 Hz, 2H, benzyl H-3, H-5), 7.22 (t, J = 7.6 Hz, 1H, H-4'), 5.19 (s, 1H, NH), 4.80 (s, 2H, benzyl CH_2), 4.29 (s, 3H, NCH_3). ^{13}C-NMR (151 MHz, $CDCl_3$) δ (ppm) 147.19 (C-7), 144.48 (C-3), 139.89 (C-4), 138.83 (benzyl C-1), 131.75 (C-1'), 129.33 (C-7a), 129.23 (benzyl C-2, C-6), 129.09 (benzyl C-4), 128.92 (benzyl C-3, C-5), 128.25 (C-3', C-5'), 127.76 (C-4'), 127.45 (C-2', C-6'), 118.47 (C-3a), 46.25 (benzyl CH_2), 39.50 (NCH_3). Anal. Calcd for $C_{19}H_{17}N_5$: C, 72.36; H, 5.43; N, 22.21. Found: C, 72.44; H, 5.52; N, 21.98.

1-Methyl-7-(morpholin-4-yl)-3-phenyl-1*H*-pyrazolo[3,4-*d*]pyridazine (**10c**): This compound was prepared following a method analogous to that of **10a**, starting from **9b**. Yield 77%. M.p.: 192-194 °C (CH_2Cl_2-Et_2O). ^{1}H-NMR (400 MHz, $CDCl_3$) δ (ppm) 9.45 (s, 1H, H-4), 7.92 (d, J = 7.3 Hz, 2H, H-2', H-6'), 7.54 (t, J = 7.3 Hz, 2H, H-3', H-5'), 7.47 (t, J = 7.3 Hz, 1H, H-4'), 4.40 (s, 3H,NCH_3), 3.99 (t, 4H, morpholine H), 3.48 (t, 4H, morpholine H). ^{13}C-NMR (151 MHz, CDCl3) δ (ppm) 152.04 (C-7) 145.57 (C-3), 143.39 (C-4), 132.52 (C-7a), 131.44 (C-1'), 129.37 (C-4'), 129.32 (C-3', C-5'), 127.54 (C-2', C-6'), 119.64 (C-3a), 66.63 (morpholine C), 51.56 (morpholine C), 38.63 (NCH_3). Anal. Calcd for $C_{16}H_{17}N_5O$: C, 65.07; H, 5.80; N, 23.71. Found: C, 64.89; H, 5.71; N, 23.86.

Ethyl 4-bromomethyl-5-isopropyl-1-methyl-1*H*-pyrazole-3-carboxylate (**11a**): This compound was prepared following a method analogous to that of **6a**, starting from **5a**. Yield 60%. Oil. ^{1}H-NMR (600 MHz, $CDCl_3$) δ (ppm) 4.79 (s, 2H, CH_2Br), 4.38 (q, J = 7.1 Hz, 2H, $C\underline{H_2}CH_3$), 3.88 (s, 3H, NCH_3), 3.19 (hept, J = 7.0 Hz, 1H, $C\underline{H}(CH_3)_2$), 1.36-1.39 (m, 9H, $CH_2C\underline{H_3}$, $CH(C\underline{H_3})_2$). ^{13}C-NMR (151 MHz, $CDCl_3$) δ (ppm) 162.22 (C=O), 148.01 (C-5), 139.48 (C-3), 118.08 (C-4), 60.95 ($\underline{C}H_2CH_3$), 38.46 (NCH_3), 25.78 ($\underline{C}H(CH_3)_2$), 23.01 (CH_2Br), 20.47 ($CH(\underline{C}H_3)_2$), 14.39 ($CH_2\underline{C}H_3$). HRMS (ESI) m/z: Calcd for $C_{11}H_{18}BrN_2O_2$: $[M+H]^+$ =289.0547, found 289.0545.

Ethyl 4-bromomethyl-1-methyl-5-phenyl-1*H*-pyrazole-3-carboxylate (**11b**): This compound was prepared following a method analogous to that of **6a**, starting from **5b**. Yield 48%. Oil. ^{1}H-NMR (400 MHz, $CDCl_3$) δ (ppm) 7.55 – 7.45 (m, 3H, H-3', H-5', H-4'), 7.42 (d, J = 7.4 Hz, 2H, H-2', H-6'), 4.56 (s, 2H, CH_2Br), 4.44 (q, J = 7.1 Hz, 2H, $C\underline{H_2}CH_3$), 3.80 (s, 3H, NCH_3), 1.41 (t, J = 7.1 Hz, 3H, $CH_2C\underline{H_3}$). ^{13}C-NMR (50 MHz, $CDCl_3$) δ (ppm) 162.01 (C=O), 144.20 (C-5), 139.90 (C-3), 129.74 (C-4'), 129.48 (C-3', C-5'), 129.07(C-2', C-6'), 127.82 (C-1'), 119.59 (C-4), 61.07 ($\underline{C}H_2CH_3$), 38.09 (NCH_3), 23.26 (CH_2Br), 14.36 ($CH_2\underline{C}H_3$). HRMS (ESI) m/z: Calcd for $C_{14}H_{16}BrN_2O_2$: $[M+H]^+$ =323.0390, found 323.0384.

Ethyl 4-formyl-5-isopropyl-1-methyl-1*H*-pyrazole-3-carboxylate(**12a)**:This compound was prepared following a method analogous to that of **7a**, starting from **11a**. Yield 61%. Oil. ^{1}H-NMR (600 MHz, $CDCl_3$) δ (ppm) 10.47 (s, 1H, CHO), 4.44 (q, J = 7.1 Hz, 2H, $C\underline{H_2}CH_3$), 3.96 (s, 3H, NCH_3), 3.65 (hept, J = 7.2 Hz, 1H, $C\underline{H}(CH_3)_2$), 1.40 (t, J = 7.1 Hz, 3H, $CH_2C\underline{H_3}$), 1.35 (d, J = 7.2 Hz, 6H, $CH(C\underline{H_3})_2$). ^{13}C NMR (151 MHz, $CDCl_3$) δ (ppm) 187.61 (CHO), 161.72 (C=O), 152.75 (C-5), 143.87 (C-3), 120.17 (C-4), 61.61 ($\underline{C}H_2CH_3$), 38.66 (NCH_3), 25.59 ($\underline{C}H(CH_3)_2$), 19.16 ($CH(\underline{C}H_3)_2$), 14.31 ($CH_2\underline{C}H_3$). HRMS (ESI) m/z: Calcd for $C_{11}H_{17}N_2O_3$: $[M+H]^+$ =225.1234, found 225.1229.

Ethyl 4-formyl-1-methyl-5-phenyl-1*H*-pyrazole-3-carboxylate (**12b**): This compound was prepared following a method analogous to that of **7a**, starting from **11b**. Yield 89%. M.p.: 79-81 °C (CH_2Cl_2-Et_2O). ^{1}H-NMR (400 MHz, $CDCl_3$) δ (ppm) 10.37 (s, 1H, CHO), 7.50 – 7.42 (m,3H, H-3', H-5', H-4'), 7.34 (d, J = 7.4 Hz,2H, H-2', H-6'), 4.46 (q, J = 7.1 Hz, 2H, $C\underline{H_2}CH_3$), 3.77 (s, 3H, NCH_3), 1.40 (t, J = 7.1 Hz, 3H, $CH_2C\underline{H_3}$). ^{13}C-NMR (50 MHz, $CDCl_3$) δ (ppm) 186.06 (CHO), 161.61 (C=O), 146.73 (C-5), 143.39 (C-3), 130.08 (C-4'), 129.77 (C-3', C-5'), 128.64 (C-2', C-6'), 127.54 (C-1'), 120.96 (C-4), 61.73 ($\underline{C}H_2CH_3$), 37.75 (NCH_3), 14.30 ($CH_2\underline{C}H_3$). HRMS (ESI) m/z: Calcd for $C_{14}H_{15}N_2O_3$: $[M+H]^+$ =259.1078, found 259.1070.

3-Isopropyl-2-methyl-2,6-dihydro-7*H*-pyrazolo[3,4-*d*]pyridazin-7-one (**13a**): This compound was prepared following a method analogous to that of **8a**, starting from **12a**. Yield 69%. M.p.: 214-216 °C (EtOAc). ^{1}H-NMR (600 MHz, $CDCl_3$) δ (ppm) 8.27 (s, 1H, H-4), 4.11 (s, 3H, NCH_3), 3.34 (hept, J = 7.0 Hz, 1H, $C\underline{H}(CH_3)_2$), 1.48 (d, J = 7.0 Hz, 6H, $CH(C\underline{H_3})_2$). ^{13}C NMR (151 MHz, $CDCl_3$) δ (ppm) 157.57 (C=O), 145.37 (C-3), 141.41 (C-7a), 133.64 (C-4), 116.12 (C-3a), 38.36 (NCH_3), 26.98 ($\underline{C}H(CH_3)_2$), 22.10 ($CH(\underline{C}H_3)_2$). HRMS (ESI) m/z: Calcd for $C_9H_{13}N_4O$: $[M+H]^+$ =193.1084, found 193.1075.

2-Methyl-3-phenyl-2,6-dihydro-7*H*-pyrazolo[3,4-*d*]pyridazin-7-one (**13b**): This compound was prepared following a method analogous to that of **8a**, starting from **12b**. Yield 68%. M.p.: 239-241 °C (EtOH). ^{1}H-NMR (600 MHz, DMSO-d_6) δ (ppm) 12.34 (s, D_2O exchang., 1H, NH), 8.09 (s, 1H, H-4), 7.70 (d, J = 7.6 Hz, 2H, H-2', H-6'), 7.62 (m, 3H, H-3', H-4', H-5'), 4.11 (s, 1H, NCH_3). ^{13}C-NMR (151 MHz, DMSO-d_6) δ (ppm) 156.43 (C=O), 140.88 (C-7a), 138.90 (C-3), 132.59 (C-4), 129.85 (C-4'), 129.53 (C-2', C-6'), 129.30 (C-3', C-5'), 127.12 (C-1'), 117.23 (C-3a), 39.10 (NCH_3). HRMS (ESI) m/z: Calcd for $C_{12}H_{11}N_4O$: $[M+H]^+$ =227.0928, found 227.0921.

7-Chloro-3-isopropyl-2-methyl-2*H*-pyrazolo[3,4-*d*]pyridazine (**14a**): This compound was prepared following a method analogous to that of **9a**, starting from **13a**. Reaction was completed in 2 h and the compound was used to the next step with no further purification.

7-Chloro-2-methyl-3-phenyl-2*H*-pyrazolo[3,4-*d*]pyridazine (**14b**): This compound was prepared following a method analogous to that of **9a**, starting from **13b**. Reaction was completed in 2 h and the compound was used to the next step with no further purification.

N-Benzyl-3-isopropyl-2-methyl-2H-pyrazolo[3,4-d]pyridazin-7-amine (**15a**):This compound was prepared following a method analogous to that of **10a**, starting from **14a**. Yield 37%. Oil. ^{1}H-NMR (600 MHz, $CDCl_3$) δ (ppm) 8.90 (s, 1H, H-4), 7.44 (d, J = 7.4 Hz, 2H, benzyl H-2, H-6), 7.33 (t, J = 7.4 Hz, 2H, benzyl H-3, H-5), 7.27 (t, J = 7.4 Hz, 1H, benzyl H-4), 4.95 (s, 2H, benzyl CH_2), 4.09 (s, 3H, NCH_3), 3.36 (hept, J = 7.2 Hz, 1H, C<u>H</u>(CH_3)$_2$), 1.50 (d, J = 7.2 Hz, 6H, CH(C<u>H$_3$</u>)$_2$). ^{13}C-NMR (151 MHz, $CDCl_3$) δ (ppm) 150.53 (C-7), 144.34 (C-3), 138.85 (benzyl C-1), 138.70 (C-4), 135.42 (C-7a), 128.74 (benzyl C-3, C-5), 128.29 (benzyl C-2, C-6), 127.58 (benzyl C-4), 114.94 (C-3a), 45.69 (benzyl CH_2), 38.34 (NCH_3), 27.22(<u>C</u>H(CH_3)$_2$), 22.12 (CH(<u>C</u>H$_3$)$_2$). *Anal.* Calcd for $C_{16}H_{19}N_5$: C, 68.30; H, 6.81; N, 24.89. Found: C, 68.58; H, 6.90; N, 24.50.

N-Benzyl-2-methyl-3-phenyl-2H-pyrazolo[3,4-d]pyridazin-7-amine (**15b**):This compound was prepared following a method analogous to that of **10a**, starting from **14b**. Yield 28%.M.p.: 211-214 °C (CH_2Cl_2-Et_2O). ^{1}H-NMR (600 MHz, $CDCl_3$) δ (ppm) 10.61 (s, 1H, H-4), 7.78 (d, J = 7.6 Hz, 2H, H-2', H-6'), 7.65 (t, J = 7.6 Hz, 2H, H-3', H-5'), 7.55 (t, J = 7.6 Hz, 1H, H-4'), 7.43 (d, J = 7.4 Hz, 2H, benzyl H-2, H-6), 7.36 (t, J = 7.4 Hz, 2H, benzyl H-3, H-5), 7.31 (t, J = 7.4 Hz, 1H, benzyl H-4), 4.78 (s, 2H, benzyl CH_2), 4.26 (s, 3H, NCH_3). ^{13}C-NMR (151 MHz, $CDCl_3$) δ (ppm) 150.88 (C-7), 146.18 (C-3), 140.49 (C-4), 136.62 (benzyl C-1), 135.03 (C-7a), 131.83 (C-4'), 130.28 (C-3', C-5'), 130.05 (C-2', C-6'), 129.10 (benzyl C-3, C-5), 128.31 (benzyl C-4), 128.27 (benzyl C-2, C-6), 124.70 (C-1'), 115.79 (C-3a), 45.69 (benzyl CH_2), 40.28 (NCH_3). *Anal.* Calcd for $C_{19}H_{17}N_5$: C, 72.36; H, 5.43; N, 22.21. Found: C, 72.61; H, 5.47; N, 21.90.

2-Methyl-7-(morpholin-4-yl)-3-phenyl-2H-pyrazolo[3,4-d]pyridazine (**15c**):This compound was prepared following a method analogous to that of **10a**, starting from **14b**. Yield 46%.M.p.: 184-186 °C (CH_2Cl_2-Et_2O). ^{1}H-NMR (400 MHz, $CDCl_3$) δ (ppm) 8.82 (s, 1H, H-4), 7.59 (m, 3H, H-3', H-5',H-4'), 7.51 (d, J = 7.2 Hz, 2H,H-2', H-6'), 4.23 (t, 4H, morpholine H), 4.19 (s, 3H, NCH_3), 3.91 (t, 4H, morpholine H). ^{13}C-NMR (151 MHz, $CDCl_3$) δ (ppm) 151.57(C-7), 139.53 (C-4), 137.81 (C-3), 136.67(C-7a), 130.07 (C-4'), 129.54 (C-2', C-6',C-3', C-5'), 127.75 (C-1'), 118.21 (C-3a), 67.20 (morpholine C), 47.00 (morpholine C), 38.93 (NCH_3). *Anal.* Calcd for $C_{16}H_{17}N_5O$: C, 65.07; H, 5.80; N, 23.71. Found: C, 65.22; H, 5.84; N, 23.55.

Pharmacological methods

Cell lines

Stable Flp-In-CHO cell lines expressing the WT A_3R were generated and maintained as previously described[13,14]. CHO-K1 cells stably expressing WT A_1R, $A_{2A}R$ or $A_{2B}R$ were routinely cultured in Hams F-12, supplemented with 10% Foetal bovine serum (FBS). All were annually checked for mycoplasma infection using an EZ-PCR mycoplasma test kit (Biological Industries, Kibbutz Beit-Haemek, Israel). Production and analysis of the mutant versions of the A_1R were as described in ref [15].

Compounds

NECA was purchased from Sigma-Aldrich and dissolved in dimethyl sulfoxide (DMSO). All compounds used for the in vitro testing were >95% pure by elemental analysis.

cAMP accumulation assay

cAMP inhibition experiments were performed using a LANCE® cAMP kit as described previously[13,14]. Briefly, Flp-In-CHO cells expressing WT A_3R and CHO-K1 cells expressing WT A_1R, $A_{2A}R$ or $A_{2B}R$ were seeded in a white 384-well optiplate at a density of 2,000 cells per well and stimulated for 30 min with a range of NECA concentrations, with or without potential antagonists, in the presence of 0.1% BSA and 25 µM rolipram, and 10 µM forskolin (to enable detection of A_1R- or A_3R-mediated inhibition of cAMP). Since the $A_{2A}R$ and $A_{2B}R$ promote cAMP accumulation, [16] the addition of forskolin was not included when assaying these receptors.

Schild analysis

NECA concentration-dependent response curves were constructed in the presence of either DMSO alone or multiple concentrations of **10b** at the A_1R and A_3R. NECA concentrations ranged from 1 pM to 1 µM. Estimates of the pEC_{50} values in the presence and absence of the antagonist were determined using the three-parameter logistic equation built into Prism.

NanoBRET assays for binding

NanoBRET competition binding assays were conducted to determine the affinity (pK_i) of various potential antagonists at the A_1R and A_3R as described previously[9]. For both the A_1R and A_3R, the CellAura fluorescent A_3R antagonist (CA200645) with a xanthine amine congener (XAC) structure was used at 20 nM and 5 nM concentration for A_1R and A_3R, respectively, since it has a slow off rate. Kinetic data was fitted with the 'kinetic of competitive binding' model (see ref.[17]; built into Prism) to determine affinity (pK_i) values and the association rate constant (K_{on}) and dissociation rates (K_{off}) for unlabelled A_3R antagonists. In agreement with our previous studies[15,18] we determined the pK_d of CA200645 at the A_1R to be 18.29 ± 2.4 nM and at the A_3R 26.95 ± 3.2 nM. [18] The BRET ratio at 10 min poststimulation was fitted with the "one-site–K_i model" derived from the Cheng and Prusoff correction, built into Prism to determine the affinity (pK_i) constant at equilibrium values for all unlabelled antagonists at the A_1R and A_3Rs.

Data and Statistical analysis

All *in vitro* assay data were analyzed using Prism 9.0 (GraphPad software, San Diego, CA), with all dose-inhibition curves being fitted using a three-parameter logistic equation to calculate response range and pEC_{50}. Experimental design ensured random distribution of treatments across 96/384-well plates to avoid systematic bias. Agonist stimulation alone was used as an intrinsic control across all experiments. Dose-inhibition/dose-response curves were normalized to forskolinstimulation ($A_{2A}R$ and $A_{2B}R$) or forskolin inhibition (A_1R and A_3R) relative to NECA (agonist allowing comparison across AR subtypes), expressed as percentage forskolin inhibition for G_i-coupled A_1R and A_3R (1 µM or 10 µM, respectively) or stimulation for $A_{2A}R$ and $A_{2B}R$ (100 µM, representing the maximum cAMP accumulation of the system), relative to NECA. For cAMP experiments on A_1R mutants, data was normalized to 100 µM forskolin, representing the maximum cAMP accumulation possible for each cell line.

Schild analysis, when using a single concentration of antagonist was performed to obtain, the dissociation constant (pK_d) using eq. (1) [19]

$$\frac{D'}{D} = 1 + [A]K_2, \qquad (1)$$

where D' and D = EC_{50} values of NECA with and without antagonist present, respectively, [A] = the concentration of antagonist present, and K_2 = the affinity constant (K_A) of the antagonist used[19]. Receptor binding kinetics was determined as described previously [18]using the Motulsky and Mahan method[17] (built into Prism 9.0) to determine the test compound association rate constant and dissociation rate constant. The data and statistical analysis comply with the recommendations on experimental design and analysis in pharmacology[20]. Statistical significance (**, $p < 0.05$;**, $p < 0.01$;***, $p < 0.001$, ;****, $p < 0.0001$)* was calculated using a one-way ANOVA with a Dunnett's post-test for multiple comparisons. All statistical analysis was performed using Prism 9.0 on data which were acquired from experiments performed a minimum of five times, conducted in duplicate.

Computational Methods

3D Similarity Calculations

All of the 3D similarity calculations were performed with Canvas program (Schrödinger Release 2021-1: Canvas, Schrödinger, LLC, New York, NY, 2021)[21]. searching in CHEMBL[21,22] database. The similar compounds structure was ranked according to the TanimotoCombo [10] coefficient as metric[24,25].

Models of the complexes between A_1R or A_3R or $A_{2B}R$ with an antagonist

We used for $A_{2A}R$ in inactive state the experimental structure of $A_{2A}R$ in complex with ZM241385 which was resolved by X-ray crystallography (PDB ID 3EML[8]). We used for A_1R in inactive state the experimental structure of A_1R in complex with DU172 which as resolved by X-ray crystallography (PDB ID 5UEN[26]). Their models were retrieved from Adenosinland web service[26].

We superimposed the experimental crystal structure ZM241385 - $A_{2A}R$ complex (PDB ID 3EML)[8] to the WT A_3R or WT $A_{2B}R$ models from Adenosiland web-service[27]. (Residue numbers in parenthesis refer to the Ballesteros–Weinstein numbering)[28]. Then, the $A_{2A}R$ (PDB ID 3EML)[8] protein was removed resulting in the WT A_3R - ZM241385 or WT $A_{2B}R$ - ZM241385 models used in the study.

In the next step, the WT A_1R - PSB36, A_3R - ZM241385, $A_{2B}R$ - ZM241385 structures were optimized using the Protein Preparation Wizard implementation in Schrodinger suite[29]. In this process, the bond orders and disulfide bonds were assigned, and missing hydrogen atoms were added. Additionally, N- and C-termini of the protein model were capped by acetyl and *N*-methyl-amino groups, respectively. All His were treated with imidazoles protonated at Nε except $H278^{7.43}$ at Nδ. Each protein was subjected in an all atom minimization using the OPLS 2005 force field[30] with heavy atom RMSD value constrained to 0.30 Å until the r.m.s of conjugate-gradient value reached < 0.05 kcal mol^{-1} $Å^{-1}$.The side chain of $V169^{5.30}$ in the WT A_3R complex was rotated to fit the conformer as was suggested[31] in order to increase the free space for the accommodation of ligands with bulky substitutions

in this area. The WT A_1R - DU172, WT A_3R - ZM241385, WT $A_{2B}R$ - ZM241385 modelswere utilized for the molecular docking calculations.

Docking calculations

The molecular docking calculations of the 6 tested 7-amino-pyrazolo[3,4-d]pyridazines (Table 1) (the protocol used for the preparation of models of the tested ligands is reported in the Supporting Information) with A_1R, A_3R, $A_{2B}R$ were performed using GOLD software[32] (GOLD Suite, Version 5.2; Cambridge Crystallographic Data Centre: Cambridge, U.K., 2015. GOLD Suite, version 5.2; Cambridge Crystallogr. Data Cent. Cambridge, U.K., 2015) and ChemScore[33] as the scoring function. The models of WT A_1R - DU172, WT A_3R - ZM241385, WT $A_{2B}R$ - ZM241385 were used as templates for the molecular docking calculations of the antagonists to the binding area of each of the receptors. Each compound was docked in the binding site of ZM241385 in the A_3R-ZM241385 model or DU172 in A_1R - DU172, model or ZM241385 in the $A_{2B}R$-ZM241385 model in an area of 15 Å around the ligand using the experimental coordinates of ZM241385 or DU172 and 20 genetic algorithm runs were applied for each docking calculation. The top-scoring docking poses were used for MD simulations to investigate the binding profile of the 6 tested 7-amino-pyrazolo[3,4-d]pyridazines (Table 1) at A_1R or A_3R or $A_{2B}R$.

MD Simulations

Each protein-ligand complex was inserted in a pre-equilibrated hydrated 1-palmitoyl-2-oleoyl-*sn*-glycero-3-phosphoethanolamine (POPE) membrane bilayer according to OPM (Orientations of Proteins in Membranes) database[34]. The orthorhombic periodic box boundaries were set 12 Å away from the protein using the System Builder utility of Desmond v4.9 (Schrödinger Release 2021-1: Desmond Molecular Dynamics System, D. E. Shaw Research, New York, NY, 2021. Maestro-Desmond Interoperability Tools, Schrödinger, New York, NY, 2021). The membrane bilayer consisted by ca. 180 lipids and 16,000 TIP3P[35] water molecules. Sodium and chloride ions were added randomly in the water phase to neutralize the system and reach the experimental salt concentration of 0.150 M NaCl. The total number of atoms of the complex was approximately 75,000 and the simulation box dimensions was (88 x 75 x 110$Å^3$). We used the Desmond Viparr tool to assign the amber99sb[36,37] force field parameters for the calculation of the protein, lipids and intermolecular interactions, and the Generalized Amber Force Field (GAFF)[38] parameters for the ligands. Ligand atomic charges were computed using the RESP[39] fitting for the electrostatic potentials calculated with Gaussian03[40] at the HF/6-31G*[41] level of theory and the antechamber of AmberTools18. [42]

100 ns MD simulations at constant pressure (NPT) were performed for the 6 ligands (Table 1) in complex with A_1R or A_3R or $A_{2B}R$ embedded in POPE bilayers using Desmond v4.9 software, the Desmond MD algorithm[43] with amber99sb[44] force field to investigate their binding interactions. The protocol used to calculate interactions and run the MD simulations and the visualization of the trajectories is described in the Supporting Information. Within the 100ns-MD simulation time, the total energy and RMSD of the protein backbone C_α atoms reached a plateau, and the systems were considered equilibrated and suitable for statistical analysis (Figure S4).

Two MD simulations were performed for each complex using the same starting structure and applying randomized velocities. All the MD simulations with Desmond software were run on GTX 1060 GPUs in lab workstations or the ARIS Supercomputer.

Computational Biochemistry Methods

Ligand preparation

The 2D structures of the 6 compounds were sketched with Marvin Program (Marvin version 21.17.0, ChemAxon (**https://www.chemaxon.com)**) and model-built with Schrödinger 2021-1 platform (Schrödinger Release 2021-1**:** Protein Preparation Wizard; Epik, Schrödinger, LLC, New York, NY, 2021; Impact, Schrödinger, LLC, New York, NY; Prime, Schrödinger, LLC, New York, NY, 2021) and minimized using the CG method, the MMFF94 force field[45] and a distance-dependent dielectric constant of 4.0 until a convergence threshold of 2.4 10^{-5} kcal mol^{-1} $Å^{-1}$ was reached. Ionization states of the compounds at pH 7.5 were tested using Epik program[46] implemented in Schrödinger suite (Prime, Schrödinger, LLC, New York, NY, 2021). Energy minimization of the compounds' 3D structures was performed using the OPLS2005[3,411,12] force field force field.

MD simulations protocol

The MD simulation of each protein-ligand complex inside the lipid bilayer was performed using the default protocol provided with Desmond v4.9 program. The MD simulation protocol consists of a series of MD simulations designed to relax the system, while not deviating substantially from the initial coordinates. During the first stage, a simulation was run for 200 ps at 10 K in the NVT ensemble (constant volume, temperature and number of atoms), with solute-heavy atoms restrained by a force constant of 50 kcal mol $Å^{-2}$. The temperature was raised to 310 K during a 200 ps MD simulation in the NPT ensemble (constant pressure, temperature and number of atoms), with the same force constant applied to the solute atoms. The temperature of 310 K was used in MD simulations in order to ensure that the membrane state is above the main phase transition temperature of 298 K for POPE bilayers[47]. The heating was then followed by equilibration simulations. First, two 1 ns stages of NPT equilibration were performed. In the first 1 ns stage, the heavy atoms of the system were restrained by applying a force constant of 10 kcal mol^{-1} $Å^{-2}$, and in the second 1 ns stage, the heavy atoms of the protein-ligand complex were restrained by applying a force constant of 2 kcal mol^{-1} $Å^{-2}$ to equilibrate water and lipid molecules. In the production phase, the relaxed systems were simulated without restraints in the NPT ensemble for 100 ns. Replicas of the system were saved every 10 ps.

In the MD simulations the Particle Mesh Ewald (PME) [48] method was employed to calculate long-range electrostatic interactions with a grid spacing of 0.8 Å. The SHAKE method was used to constrain heavy atom-hydrogen bonds at ideal lengths and angles[49]. Van der Waals and short-range electrostatic interactions were smoothly truncated at 12 Å[50]. The Nosé-Hoover thermostat[51] was utilized to maintain a constant temperature in all MD simulations, and the Martyna-Tobias-Klein method[52] was used to control the pressure. The equations of motion were integrated using the multistep reversible reference system propagator algorithms (RESPA)[53] integrator with an inner time step of 2 fs for bonded

interactions and non-bonded interactions within the cutoff of 12 Å. An outer time step of 6.0 fs was used for non-bonded interactions beyond the cutoff.

The visualization of the MD simulation trajectories was performed using the graphical user interface (GUI) of Maestro and the protein-ligand interaction analysis was carried out with the Simulation Interaction Diagram (SID) tool, available with Desmond v4.9 program. For the calculation of hydrogen bond interactions were considered, a distance 2.5 Å between donor and acceptor heavy atoms, and angle $\geq 120^{o}$ between donor-hydrogen-acceptor atoms and $\geq 90^{o}$ between hydrogen-acceptor-bonded atom. Non-specific hydrophobic contacts were identified when the side chain of a hydrophobic residue fell within 3.6 Å from a ligand's aromatic or aliphatic carbon, while π-π interactions were characterized by stacking of two aromatic groups face-to-face or face-to-edge. Water-mediated interactions were characterized when the distance between donor and acceptor atoms was 2.7 Å, as well as an angle $\geq 110^{o}$ between donor-hydrogen-acceptor atoms and $\geq 80^{o}$ between hydrogen-acceptor-bonded atom.

MM-GBSA calculations

The MD simulation trajectories were used for the calculation of approximate binding free energies of ligand – protein complexes using the 1-trajectory MM-GBSA[3,3] method, the OPLS2005[3,4] force field and 20 waters in the vicinity of the ligand . The MD trajectories were processed with the Python library MDAnalysis 54[13] in order to extract the 20 water molecules closest to any atom in the ligand 55[14] for each of the 501 frames. We applied a dielectric constant $\varepsilon_{\text{solute}}$ = 1 to the binding area and to account for the lipophilic environment of the protein an heterogeneous dielectric implicit membrane model was used along the bilayer z-axis[39].

For this, structural ensembles were extracted in intervals of 40 ps from the 20 ns MD simulation for each complex. Prior to the calculations all water molecules, ions, and lipids were removed, except 20 waters in the vicinity of the ligand[55], and the structures were positioned such that the geometric centre of each complex was located at the coordinate origin.The MD trajectories were processed with the Python library MDAnalysis [53] in order to extract the 20 water molecules closest to any atom in the ligand for each of the 501 frames. During the MM-GBSA calculations, the explicit water molecules were considered as being part of the protein. Binding free energies of compounds in complex with A_1R and A_3R were estimated using the 1-trajectory MM-GBSA approach. [3,31^{15,16}^{15,16}]For the calculation of binding free energy for each complex eqs (1)-(4) were used

$$\Delta G_{\text{bind}} = \langle G_{\text{complex}} - G_{\text{protein}} - G_{\text{ligand}} \rangle_{\text{complex}} \quad (1)$$

$$G_i = V_{\text{MM}} - T\langle S_{\text{MM}} \rangle + \Delta G_{\text{solv}} \quad (2)$$

$$V_{\text{MM}} = V_{\text{bonded}} + V_{\text{coul}} + V_{\text{LJ}} \quad (3)$$

$$\Delta G_{\text{solv}} = \Delta G_{\text{P}} + \Delta G_{\text{NP}} \quad (4)$$

The binding free energy for each complex was thus calculated using eq. (5)

$$\Delta G_{\text{bind}} = \langle \Delta E_{\text{coul}} + \Delta E_{LJ} \rangle - T\langle \Delta S_{\text{MM}} \rangle + \Delta\Delta G_{\text{solv}} \quad (5)$$

In eqs (1)-(4) G_i is the free energy of system *i*, that being the ligand, the protein, or the complex; V_{MM} is the potential energy in vacuum as defined by the molecular mechanics (MM) model, which is composed of the bonded potential energy terms (V_{bonded}) and nonbonded Coulombic (V_{coul}) and Lennard-Jones (V_{LJ}) terms; S_{MM} is the entropy; ΔG_{solv} is the free energy of solvation for transferring the ligand from water in the binding area calculated using the PBSA model, composed by a polar (ΔG_P) and nonpolar (ΔG_{NP}) term; *T* is the temperature and angle brackets represent an ensemble average. Molecular mechanics energies for Lennard-Jones (V_{LJ}) and Coulombic elecrostatic (V_{coul}) were calculated with OPLS2005 [3,4] force field ; in these calculations $\Delta V_{bonded} = 0$ as the single trajectory method was adopted and $\Delta V_{MM} = \Delta V_{LJ}$ and ΔV_{coul}.The polar part of the solvation free energy was determined by calculations using the Generalized-Born model. 54[17] The nonpolar term was considered proportional to the solvent accessible surface area (*SASA*), $\Delta G_{NP} = \gamma \cdot SASA$, where $\gamma = 0.0227$ kJ mol^{-1} Å^{-2}. The entropy term was neglected since we were interested for binding free energies between ligands in the same series and in this case ΔG_{bind} is termed as effective binding energy, ΔG_{eff}. The post-processing thermal_mmgbsa.py script of the Schrodinger Suite was used which takes snapshots from the MD simulations trajectory and calculates ΔG_{eff}.

Cell viability assays

Human HCT116 colon cancer cell line and PC-3 prostate cancer cell linewere obtained from the American Type Cell Culture (ATCC, Bethesda, Md). Both cell lines were grown in 75-cm^2 culture flasks at 37°C in 5% CO_2 using Roswell Park Memorial Institute 1640 medium (RPMI 1640, Gibco) and Dulbecco's modified Eagle's medium F/12 (DMEM/F-12, Gibco) respectively, containing 10% fetal bovine serum (FBS, Gibco). To test the inhibitory activities of compounds using a cell-based assay, HCT116 cells were plated at a density of 1500 per well, while PC-3 cells were plated at a density of 750 per well in a 96-well plate. After 24 h, cells were treated with the indicated compounds in a dose-dependent manner for 72h and 96h (all tested compounds provided clear solutions). MTT [3-(4,5-dimethylthiazol-2-yl)-2,5-diphenyltetrazolium bromide] (Sigma M-5655) was added at a final concentration of 0.5 mg/mL directly to each well for 4 hours at 37°C. The medium was aspirated and the blue MTT formazan precipitate was dissolved in dimethyl sulfoxide (DMSO). Absorbance was determined in a Powerwave microplate spectrophotometer (Biotek Instruments, Inc.) at 540 nm. Viable cell numbers were determined by tetrazolium conversion to its formazan dye. The IC_{50} was calculated by Microsoft Excel equation and confirmed by GraphPad Prism (7.0). Each experiment was performed in triplicate and mean values ± SD are reported.

Abbreviations

AcOEt, ethyl acetate; CHO, Chinese hamster ovary; COPD, chronic obstructive pulmonary disease; CPT, 8-cyclopentyl-1,3-dimethylxanthine; cryo-em, cryogenic electron microscopy; 2D NMR, two-dimensional NMR, EL2, Extracellular loop 2; MAPK, mitogen-activated protein kinase; OPM, Orientations of Proteins in Membranes; PD, Parkinson's diseasePME, particle mesh Ewald method; POPC, 1-palmitoyl-2-oleoyl-*sn*-glycero-3-phosphocholine; RESPA, Reversible multiple time scale molecular dynamics.

References

(1) Ballesteros, J. A.; Weinstein, H. Analysis and Refinement of Criteria for Predicting the Structure and Relative Orientations of Transmembranal Helical Domains. *Biophys. J.* **1992**.

(2) Barkan, K.; Lagarias, P.; Stampelou, M.; Stamatis, D.; Hoare, S.; Safitri, D.; Klotz, K.-N. N.; Vrontaki, E.; Kolocouris, A.; Ladds, G. Pharmacological Characterisation of Novel Adenosine A3 Receptor Antagonists. *Sci. Rep.* **2020**, *10*, 20781.

(3) Massova, I.; Kollman, P. A. *Combined Molecular Mechanical and Continuum Solvent Approach (MM- PBSA/GBSA) to Predict Ligand Binding*; 2000; Vol. 18, pp. 113–135.

(4) Jaakola, V.-P.; Griffith, M. T.; Hanson, M. A.; Cherezov, V.; Chien, E. Y. T.; Lane, J. R.; IJzerman, A. P.; Stevens, R. C. The 2.6 Angstrom Crystal Structure of a Human A2A Adenosine Receptor Bound to an Antagonist. *Science (80-.).* **2008**, *322*, 1211–1217.

(5) Manetti, F.; Schenone, S.; Bondavalli, F.; Brullo, C.; Bruno, O.; Ranise, A.; Mosti, L.; Menozzi, G.; Fossa, P.; Trincavelli, M. L.; Martini, C.; Martinelli, A.; Tintori, C.; Botta, M. Synthesis and 3D QSAR of New Pyrazolo[3,4-b]Pyridines: Potent and Selective Inhibitors of A1 Adenosine Receptors. *J. Med. Chem.* **2005**.

(6) Thompson, R. D.; Olsson, R. A.; Secunda, S.; Daly, J. W.; Olsson, R. A. N6,9-Disubstituted Adenines: Potent, Selective Antagonists at the A1 Adenosine Receptor. *J. Med. Chem.* **1991**.

(7) Havlíček, L.; Hanuš, J.; Veselý, J.; Leclerc, S.; Meijer, L.; Shaw, G.; Strnad, M. Cytokinin-Derived Cyclin-Dependent Kinase Inhibitors: Synthesis and Cdc2 Inhibitory Activity of Olomoucine and Related Compounds. *J. Med. Chem.* **1997**.

(8) Glukhova, A.; Thal, D. M.; Nguyen, A. T.; Vecchio, E. A.; Jörg, M.; Scammells, P. J.; May, L. T.; Sexton, P. M.; Christopoulos, A. Structure of the Adenosine A1Receptor Reveals the Basis for Subtype Selectivity. *Cell* **2017**, *168*, 867–877.

(9) Barkan, K.; Lagarias, P.; Stampelou, M.; Stamatis, D.; Hoare, S.; Safitri, D.; Klotz, K.-N.; Vrontaki, E.; Kolocouris, A.; Ladds, G. Pharmacological Characterisation of Novel Adenosine A3 Receptor Antagonists. *Sci. Rep.* **2020**, *10*, 20781.

(10) Hawkins, P. C. D.; Skillman, A. G.; Nicholls, A. Comparison of Shape-Matching and Docking as Virtual Screening Tools. *J. Med. Chem.* **2007**, *50*, 74–82.

(11) Kaminski, G. A.; Friesner, R. A.; Tirado-Rives, J.; Jorgensen, W. L. Evaluation and Reparametrization of the OPLS-AA Force Field for Proteins via Comparison with Accurate Quantum Chemical Calculations on Peptides. *J. Phys. Chem. B* **2001**, *105*, 6474–6487.

(12) Shivakumar, D.; Williams, J.; Wu, Y. J.; Damm, W.; Shelley, J.; Sherman, W. Prediction of Absolute Solvation Free Energies Using Molecular Dynamics Free Energy Perturbation and the OPLS Force Field. *J. Chem. Theory Comput.* **2010**, *6*, 1509–1519.

(13) Michaud-Agrawal, N.; Denning, E. J.; Woolf, T. B.; Beckstein, O. MDAnalysis: A Toolkit for the Analysis of Molecular Dynamics Simulations. *J. Comput. Chem.* **2011**, *32*, 2319–2327.

(14) Maffucci, I.; Contini, A. Explicit Ligand Hydration Shells Improve the Correlation between MM-PB/GBSA Binding Energies and Experimental Activities. *J. Chem. Theory Comput.* **2013**, *9*, 2706–2717.

(15) Genheden, S.; Ryde, U. The MM/PBSA and MM/GBSA Methods to Estimate Ligand-Binding Affinities. *Expert Opin. Drug Discov.* **2015**, *10*, 449–461.

(16) Homeyer, N.; Gohlke, H. Free Energy Calculations by the Molecular Mechanics Poisson-Boltzmann Surface Area Method. *Mol. Inform.* **2012**, *31*, 114–122.

(17) Qiu, D.; Shenkin, P. S.; Hollinger, F. P.; Still, W. C. The GB/SA Continuum Model for Solvation. A Fast Analytical Method for the Calculation of Approximate Born Radii. *J.*

Phys. Chem. A **1997**, *101*, 3005–3014.

(11) Yan, Z.; Liu, A.; Huang, M.; Liu, M.; Pei, H.; Huang, L.; Yi, H.; Liu, W.; Hu, A. Design, Synthesis, DFT Study and Antifungal Activity of the Derivatives of Pyrazolecarboxamide Containing Thiazole or Oxazole Ring. *Eur. J. Med. Chem.***2018**, *149*, 170–181.

(12) Schmidt, A.; Habeck, T.; Kindermann, M. K.; Nieger, M. New Pyrazolium-Carboxylates as Structural Analogues of the Pseudo-Cross-Conjugated Betainic Alkaloid Nigellicine. *J. Org. Chem.***2003**, *68*, 5977–5982.

(13) Knight, A.; Hemmings, J. L.; Winfield, I.; Leuenberger, M.; Frattini, E.; Frenguelli, B. G.; Dowell, S. J.; Lochner, M.; Ladds, G. Discovery of Novel Adenosine Receptor Agonists That Exhibit Subtype Selectivity. *J. Med. Chem.***2016**, *59*, 947–964.

(14) Weston, C.; Poyner, D.; Patel, V.; Dowell, S.; Ladds, G. Investigating G Protein Signalling Bias at the Glucagon-like Peptide-1 Receptor in Yeast. *Br. J. Pharmacol.***2014**, *171*, 3651–3665.

(15) Deganutti, G.; Barkan, K.; Ladds, G.; Reynolds, C. A. Multisite Model of Allosterism for the Adenosine A1 Receptor. *J. Chem. Inf. Model.***2021**, *61*, 2001–2015.

(16) Zhang, G.; Liu, Y.; Ruoho, A. E.; Hurley, J. H. Structure of the Adenylyl Cyclase Catalytic Core. *Nature***1997**, *386*, 247–253.

(17) Motulsky, H. J.; Mahan, L. C. The Kinetics of Competitive Radioligand Binding Predicted by the Law of Mass Action. *Mol. Pharmacol.***1984**.

(18) Barkan, K.; Lagarias, P.; Stampelou, M.; Stamatis, D.; Hoare, S.; Safitri, D.; Klotz, K.-N.; Vrontaki, E.; Kolocouris, A.; Ladds, G. Pharmacological Characterisation of Novel Adenosine A3 Receptor Antagonists. *Sci. Rep.***2020**, *10*, 20781.

(19) Tallarida, R. J.; Murray, R. B. *Manual of Pharmacologic Calculations*; Springer New York: New York, NY, 1986.

(20) Curtis, M. J.; Alexander, S.; Cirino, G.; Docherty, J. R.; George, C. H.; Giembycz, M. A.; Hoyer, D.; Insel, P. A.; Izzo, A. A.; Ji, Y.; MacEwan, D. J.; Sobey, C. G.; Stanford, S. C.; Teixeira, M. M.; Wonnacott, S.; Ahluwalia, A. Experimental Design and Analysis and Their Reporting II: Updated and Simplified Guidance for Authors and Peer Reviewers. *British Journal of Pharmacology*, 2018, *175*, 987–993.

(21) Duan, J.; Dixon, S. L.; Lowrie, J. F.; Sherman, W. Analysis and Comparison of 2D Fingerprints: Insights into Database Screening Performance Using Eight Fingerprint Methods. *J. Mol. Graph. Model.***2010**, *29*, 157–170.

(22) Gaulton, A.; Bellis, L. J.; Bento, A. P.; Chambers, J.; Davies, M.; Hersey, A.; Light, Y.; McGlinchey, S.; Michalovich, D.; Al-Lazikani, B.; Overington, J. P. ChEMBL: A Large-Scale Bioactivity Database for Drug Discovery. *Nucleic Acids Res.***2012**, *40*, D1100-7.

(23) Bento, A. P.; Gaulton, A.; Hersey, A.; Bellis, L. J.; Chambers, J.; Davies, M.; Krüger, F. A.; Light, Y.; Mak, L.; McGlinchey, S.; Nowotka, M.; Papadatos, G.; Santos, R.; Overington, J. P. The ChEMBL Bioactivity Database: An Update. *Nucleic Acids Res.***2014**, *42*, D1083–D1090.

(24) Hawkins, P. C. D.; Skillman, A. G.; Nicholls, A. Comparison of Shape-Matching and Docking as Virtual Screening Tools. *J. Med. Chem.***2007**, *50*, 74–82.

(25) Fernández-De Gortari, E.; García-Jacas, C. R.; Martinez-Mayorga, K.; Medina-Franco, J. L. Database Fingerprint (DFP): An Approach to Represent Molecular Databases. *J. Cheminform.***2017**.

(26) Glukhova, A.; Thal, D. M.; Nguyen, A. T.; Vecchio, E. A.; Jörg, M.; Scammells, P. J.; May, L. T.; Sexton, P. M.; Christopoulos, A. Structure of the Adenosine A1 Receptor Reveals the Basis for Subtype Selectivity. *Cell***2017**, *168*, 867-877.e13.

(27) Floris, M.; Sabbadin, D.; Medda, R.; Bulfone, A.; Moro, S. Adenosiland: Walking through Adenosine Receptors Landscape. *Eur. J. Med. Chem.***2012**, *58*, 248–257.

(28) Ballesteros, J. A.; Weinstein, H. [19] Integrated Methods for the Construction of Three-

Dimensional Models and Computational Probing of Structure-Function Relations in G Protein-Coupled Receptors. In; Elsevier, 1995; Vol. 25, pp. 366–428.
(29) Protein Preparation Wizard, Epik Version 2.2, Impact Version 5.7, Prime Version 3.0, Schrödinger, LLC., New York, NY. *Protein Prep. Wizard 2015-2; Epik version 2.4, Schrödinger, LLC, New York, NY, 2015; Impact version 5.9, Schrödinger, LLC, New York, NY, 2015; Prime version 3.2, Schrödinger, LLC, New York, NY, 2015***2011**.
(30) Kaminski, G. A.; Friesner, R. A.; Tirado-Rives, J.; Jorgensen, W. L. Evaluation and Reparametrization of the OPLS-AA Force Field for Proteins via Comparison with Accurate Quantum Chemical Calculations on Peptides. *J. Phys. Chem. B***2001**, *105*, 6474–6487.
(31) Katritch, V.; Kufareva, I.; Abagyan, R. Structure Based Prediction of Subtype-Selectivity for Adenosine Receptor Antagonists. *Neuropharmacology***2011**, *60*, 108–115.
(32) Jones, G.; Willett, P.; Glen, R. C.; Leach, A. R.; Taylor, R. Development and Validation of a Genetic Algorithm for Flexible Docking. *J. Mol. Biol.***1997**, *267*, 727–748.
(33) Eldridge, M. D.; Murray, C. W.; Auton, T. R.; Paolini, G. V.; Mee, R. P. Empirical Scoring Functions: I. The Development of a Fast Empirical Scoring Function to Estimate the Binding Affinity of Ligands in Receptor Complexes. *J. Comput. Aided. Mol. Des.***1997**, *11*, 425–445.
(34) Lomize, M. A.; Pogozheva, I. D.; Joo, H.; Mosberg, H. I.; Lomize, A. L. OPM Database and PPM Web Server: Resources for Positioning of Proteins in Membranes. *Nucleic Acids Res.***2012**, *40*, D370–D376.
(35) Jorgensen, W. L.; Chandrasekhar, J.; Madura, J. D.; Impey, R. W.; Klein, M. L. Comparison of Simple Potential Functions for Simulating Liquid Water. *J. Chem. Phys.***1983**, *79*, 926–935.
(36) Wang, J.; Cieplak, P.; Kollman, P. A. How Well Does a Restrained Electrostatic Potential (RESP) Model Perform in Calculating Conformational Energies of Organic and Biological Molecules? *J. Comput. Chem.***2000**, *21*, 1049–1074.
(37) Hornak, V.; Abel, R.; Okur, A.; Strockbine, B.; Roitberg, A.; Simmerling, C. Comparison of Multiple Amber Force Fields and Development of Improved Protein Backbone Parameters. *Proteins Struct. Funct. Genet.***2006**, *65*, 712–725.
(38) Wang, J.; Wolf, R. M.; Caldwell, J. W.; Kollman, P. A.; Case, D. A. Development and Testing of a General Amber Force Field. *J. Comput. Chem.***2004**, *25*, 1157–1174.
(39) Bayly, C. I.; Cieplak, P.; Cornell, W. D.; Kollman, P. A. A Well-Behaved Electrostatic Potential Based Method Using Charge Restraints for Deriving Atomic Charges: The RESP Model. *J. Phys. Chem.***1993**, *97*, 10269–10280.
(40) Frisch, M. J.; Trucks, G. W.; Schlegel, H. B.; Scuseria, G. E.; Robb, M. A.; Cheeseman, J. R.; Montgomery, J. A., Jr.; Vreven, T.; Kudin, K. N.; Burant, J. C.; Millam, J. M.; Iyengar, S. S.; Tomasi, J.; Barone, V.; Mennucci, B.; Cossi, M.; Scalmani, G.; Re, J. A. Gaussian 03. **2007**.
(41) Davidson, E. R.; Feller, D. Basis Set Selection for Molecular Calculations. *Chem. Rev.***1986**, *86*, 681–696.
(42) D.A. Case; I.Y. Ben-Shalom; S.R. Brozell; D.S. Cerutti; T.E. Cheatham; III, V. W. D. C.; T.A. Darden; R.E. Duke; D. Ghoreishi; M.K. Gilson; H. Gohlke; A.W. Goetz; D. Greene; R Harris; N. Homeyer; S. Izadi; A. Kovalenko; T. Kurtzman; T.S. Lee; S. LeGrand; P. Li; C. Lin; J. Liu; T. Luchko; R. Luo; D.J. Mermelstein; K.M. Merz; Y. Miao; G. Monard; C. Nguyen; H. Nguyen; I. Omelyan; A. Onufriev; F. Pan; R. Qi; D.R. Roe; A. Roitberg; C. Sagui; S. Schott-Verdugo; J. Shen; C.L. Simmerling; J. Smith; R. Salomon-Ferrer; J. Swails; R.C. Walker; J. Wang; H. Wei; R.M. Wolf; X. Wu; L. Xiao; D.M. York; P.A. Kollman. *AMBER 2018, University of California*; 2018.
(43) Bowers, K. J.; Chow, E.; Xu, H.; Dror, R. O.; Eastwood, M. P.; Gregersen, B. A.; Klepeis, J. L.; Kolossvary, I.; Moraes, M. A.; Sacerdoti, F. D.; Salmon, J. K.; Shan, Y.;

Shaw, D. E. Scalable Algorithms for Molecular Dynamics Simulations on Commodity Clusters. In *Proceedings of the 2006 ACM/IEEE Conference on Supercomputing, SC'06*; ACM Press: New York, New York, USA, New York, USA, 2006; p. 84.
(44) Kim, J.; Jiang, Q.; Glashofer, M.; Yehle, S.; Wess, J.; Jacobson, K. A. Glutamate Residues in the Second Extracellular Loop of the Human A2a Adenosine Receptor Are Required for Ligand Recognition. *Mol. Pharmacol.***1996**, *49*, 683–691.
(45) Shelley, J. C.; Cholleti, A.; Frye, L. L.; Greenwood, J. R.; Timlin, M. R.; Uchimaya, M. Epik: A Software Program for PK a Prediction and Protonation State Generation for Drug-like Molecules. *Journal of Computer-Aided Molecular Design*, 2007, *21*, 681–691.
(46) Koynova, R.; Caffrey, M. Phases and Phase Transitions of the Phosphatidylcholines. *Biochim. Biophys. Acta - Rev. Biomembr.***1998**, *1376*, 91–145.
(47) Darden, T.; York, D.; Pedersen, L. Particle Mesh Ewald: An Nlog(N) Method for Ewald Sums in Large Systems. *J. Chem. Phys.***1993**, *98*, 10089–10092.
(48) Essmann, U.; Perera, L.; Berkowitz, M. L.; Darden, T.; Lee, H.; Pedersen, L. G. A Smooth Particle Mesh Ewald Method. *J. Chem. Phys.***1995**, *103*, 8577–8593.
(49) Balusek, C.; Hwang, H.; Lau, C. H.; Lundquist, K.; Hazel, A.; Pavlova, A.; Lynch, D. L.; Reggio, P. H.; Wang, Y.; Gumbart, J. C. Accelerating Membrane Simulations with Hydrogen Mass Repartitioning. *J. Chem. Theory Comput.***2019**, *15*, 4673–4686.
(50) Martyna, G. J.; Klein, M. L.; Tuckerman, M. Nosé–Hoover Chains: The Canonical Ensemble via Continuous Dynamics. *J. Chem. Phys.***1992**, *97*, 2635–2643.
(51) Martyna, G. J.; Tobias, D. J.; Klein, M. L. Constant Pressure Molecular Dynamics Algorithms. *J. Chem. Phys.***1994**, *101*, 4177–4189.
(52) Humphreys, D. D.; Friesner, R. A.; Berne, B. J. A Multiple-Time-Step Molecular Dynamics Algorithm for Macromolecules. *J. Phys. Chem.***1994**, *98*, 6885–6892.
(53) Michaud-Agrawal, N.; Denning, E. J.; Woolf, T. B.; Beckstein, O. MDAnalysis: A Toolkit for the Analysis of Molecular Dynamics Simulations. *J. Comput. Chem.***2011**, *32*, 2319–2327.
(54) Maffucci, I.; Contini, A. Explicit Ligand Hydration Shells Improve the Correlation between MM-PB/GBSA Binding Energies and Experimental Activities. *J. Chem. Theory Comput.***2013**, *9*, 2706–2717.
(55) Qiu, D.; Shenkin, P. S.; Hollinger, F. P.; Still, W. C. The GB/SA Continuum Model for Solvation. A Fast Analytical Method for the Calculation of Approximate Born Radii. *J. Phys. Chem. A***1997**, *101*, 3005–3014.
(54) Holger, G.; A., C. D. Converging Free Energy Estimates: MM-PB(GB)SA Studies on the Protein–Protein Complex Ras–Raf. *J. Comput. Chem.***2004**, *25*, 238–250.
